# Mini-Review: Stemflow as a Resource Limitation to Near-Stem Soils

**DOI:** 10.3389/fpls.2018.00248

**Published:** 2018-02-27

**Authors:** John T. Van Stan, Dennis A. Gordon

**Affiliations:** Geology and Geography, Georgia Southern University, Statesboro, GA, United States

**Keywords:** ecohydrology, stemflow, forest ecology, plant–soil interactions, arid environment

## Abstract

Stemflow, a precipitation and solute supply to soils near tree stems, can play a wide array of roles in ecosystem functioning. However, stemflow’s ecohydrological functions have been primarily studied in forests with voluminous stemflow because resource subsidy is currently considered stemflow’s only impact on near-stem soils. This common assumption ignores controls that stemflow generation may exert via resource limitation (when stemflow < open rainfall and near-stem throughfall is negligible). We reviewed selected literature across numerous forests to evaluate the predominance of stemflow as a potential resource limitation to near-stem soils and characterized the concentrated, but meager, solute flux from low stemflow generators. Global observations of stemflow were highly skewed (skewness = 4.6) and leptokurtic (kurtosis = 28.8), where 69% of observations were ≤2% of rainfall. Stemflow ≤ 2% of rainfall is 10–100 times more chemically enriched than open rainfall, yet low volumes result in negligible solute fluxes (under 1 g m^-2^ y^-1^). Reduced stemflow may be the global and regional norm, creating persistently dry near-stem soils that receive infrequent, salty, and paltry precipitation flux if throughfall is also low. Ignoring stemflow because it results in scarcity likely limits our understanding of ecosystem functioning as resource limitations alter the fate of soil nutrients, energy flows, and spatial patterning of biogeochemical processes.

## Introduction: Stemflow Feasts and Famines

**FIGURE 1:**
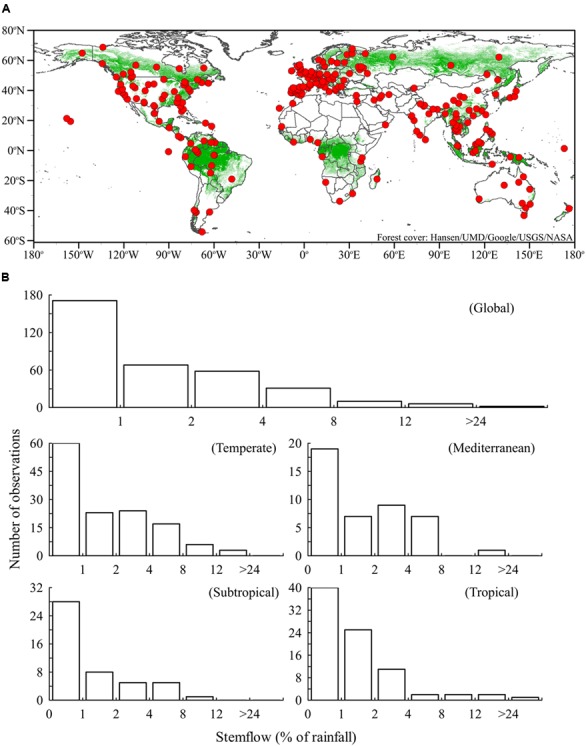
**(A)** Locations around the globe (Supplementary Table S1) where stemflow has been measured are well-distributed throughout the Earth’s forests. **(B)** Histograms of stemflow (% rainfall) from studies reporting on 346 tree species or forest types (Supplementary Table S2). Studies that simply report “negligible” stemflow have been placed in the <1% category in the histogram.

Forest canopies are often the first terrestrial surfaces to store, evaporate and redistribute precipitation, thereby affecting all subsequent hydrological processes (Savenije, 2004) and related energy flows (Davies-Barnard et al., 2014). Precipitation that is redistributed to the forest surface either drips through canopy gaps and from canopy surfaces, as throughfall, or drains along tree stems, as stemflow. This precipitation redistribution produces persistent patterns of wet and dry areas at the forest floor (Keim et al., 2005; Johnson and Lehmann, 2006) that influence soil physicochemical and microbial community characteristics (Chang and Matzner, 2000; Moore et al., 2016; Rosier et al., 2016). Most rainfall is redistributed as throughfall (>70%), generally resulting in <5% of rainfall becoming stemflow (Levia and Frost, 2003). However, stemflow from 5% of rainfall across the area of a mature tree canopy can funnel >20 times more water to near-stem soils than open rainfall on an equivalent area (Rodrigo et al., 2003). This stemflow “funneling” effect has been reviewed extensively (Levia and Frost, 2003; Johnson and Lehmann, 2006; Ikawa, 2007; McKee, 2010; Levia and Germer, 2015) and, in part, inspired stemflow studies around the globe (**Figure 1A**). Yet, instances where the funneling effect fails (stemflow inputs < throughfall or open rainfall) are rarely discussed, often ignored, and, as a result, the authors know of no study that has placed reduced stemflow in biogeochemical context. Low stemflow generation may produces the opposite extreme of the funneling effect if poorly subsidized by near-stem throughfall—localized resource scarcity—which is a condition with profound impacts on soil biogeochemical players and processes (Manzoni et al., 2012).

For example, soils surrounding the stems of Amazonian *Eschweilera* trees receive 0.07% of the annual rainfall, with the remaining rainfall becoming throughfall or returned to the atmosphere via interception (Schroth et al., 2001). This results in 127 L of rain each year reaching an infiltration area around individual *Eschweilera* stems ≥0.8 m^2^ (Schroth et al., 2001). Consequently, if throughfall is absent or low near the stem, the stemflow process surprisingly classifies tropical rainforest soils near *Eschweilera* stems as “highly arid” per the De Martonne Aridity Index (4.4 mm °C^-1^) based on the reported mean annual temperature (26°C). Near-stem soils of other tropical rainforest tree species studied by Schroth et al. (2001) also classify as arid-to-semiarid based on low stemflow supply: *Bixa orellana* (4.6 mm °C^-1^) and *Theobroma grandiflorum* (12.0 mm °C^-1^). Furthermore, the classical definition of “true deserts” (mean annual precipitation < 200 mm y^-1^: Ward, 2015) classifies the ∼160 mm y^-1^ of stemflow precipitation reaching soils near the stems of these tropical rainforest tree species as arid. The juxtaposition of two extreme stemflow conditions (stemflow funneling versus scarcity) raises questions not addressed by previous reviews: what is the frequency of low versus high stemflow observations in forests across the globe? Moreover, what is the shape of the distribution of global stemflow observations in forests? Previous work has reported mean and median stemflow production values across climates (Levia and Frost, 2003; McKee, 2010), but past stemflow statistics were not controlled for repetitive sampling of forests/species in areas with greater research output (e.g., European and northeastern United States hardwoods), nor does previous work report how well a median or mean represented the data distribution (i.e., was the data skewed and, if so, to what degree?).

How stemflow production varies across forest systems has important biogeochemical implications beyond water abundance or scarcity for near-stem soils. The concentration and flux of solutes, particulates, microbial cells, and fungal spores transported by stemflow to the surface are strongly linked to a canopy’s ability to efficiently drain rainfall to the stem (Levia and Germer, 2015). In this way, stemflow quantity is linked to soil structure (Li et al., 2009), soil chemistry (Aboal et al., 2015), soil solution chemistry (Chang and Matzner, 2000), and soil microbial community structure (Rosier et al., 2016). This mini-review evaluated select literature that represents unique forest type-climate combinations around the globe to test the primary hypothesis that stemflow, globally and across major climate zones, may be more often a resource limitation than a resource subsidy to forests’ near-stem soils. Next, we review the chemical concentration, flux, and edaphic effects of low stemflow generation to describe the unique near-stem soil conditions it engenders. The review and contextualization of a major canopy influence on near-stem soil areas in forests strengthens our understanding of canopy-soil interchanges, many of which are linked to global biogeochemical processes of societal significance (Bonan, 2016).

## Global Observations Indicate Stemflow Scarcity may be the Norm

To date, stemflow has been sampled in most forest ecosystems (**Figure 1A**, data provided in Supplementary Table S1), from *Nothofagus pumilio* at the southern-most tip of Patagonia (54°S: Frangi et al., 2005) to *Picea glauca* in the Northwest Territories (69° N: Gill, 1975). Stemflow has also been measured in several remote ecosystems, including the Galapagos Islands (Pryet et al., 2012), Canary Islands (Aboal et al., 2015), Central Pacific atolls of the Republic of Kiribati (White et al., 2007), and the Namibian Desert (Roth-Nebelsick et al., 2012) (**Figure 1A**). An important data gap remains in the Congo tropical rainforest (**Figure 1A**), a biodiversity hotspot that plays important roles in global biogeochemical cycles. Stemflow research in the boreal forests has been sparse, particularly in Russia (**Figure 1A**). In contrast, stemflow of northern temperate forests has been extensively studied (**Figure 1A**).

A survey of 346 observations selected to represent the range of combinations in tree species-climate conditions (Supplementary Table S2) indicates that approximately two-thirds (69%) of stemflow observations fall below 2% of rainfall (**Figure 1B**). Drainage of less than 2% of rainfall across a tree canopy can have a range of outcomes regarding precipitation supply to the near-stem soils dependent, in large part, on the ratio of the canopy area to near-stem infiltration area. For stemflow produced from 2% of rainfall to equal open rainwater supply, for example, the ratio of canopy area (for capturing and draining water to the stem) to a conservative 1 m^2^ infiltration area around the stem base (this area is unlikely to be smaller for most mature trees) would have to be 50:1. Thus, forests draining ≤2% of rainfall as stemflow are likely depriving near-stem soils of water relative to the open if near-stem throughfall is negligible. Despite this, most attention regarding stemflow and its biogeochemical impacts is focused on the rarer instances where stemflow is voluminous (Levia and Germer, 2015). Of course, stemflow percentage widely ranged across species, with a minimum of 0.003% for *Larix cajanderi* in Siberia (Toba and Ohta, 2005) and maximum of 33.9% for *Psidium cattleyanum* invading Hawaiian forests (Safeeq and Fares, 2012).

The data distributions for global and, when enough observations were available, individual climates were strongly skewed (**Figure 1B**). Shape of the data distributions were analyzed for skewness (asymmetry) and kurtosis (tail structure). A common rule for kurtosis is that normal distributions are <3 (platykurtic) while long, “heavy” tailed distributions are >3 (leptokurtic). Skewness of global stemflow data was double the common normality threshold (4.6 ± 0.1 SE versus 2.0) and it was strongly leptokurtic (28.8 ± 0.3 SE). Therefore, mean (and even median) stemflow percentage does not represent the data’s central tendency well. Mean and median stemflow values have been reported, ranging 3.7–5.0%, 3.5–11.3%, 1.6–4.0% for boreal, temperate, and tropical forests, respectively (Levia and Frost, 2003; McKee, 2010), but our literature synthesis reveals that a majority of stemflow observations in boreal, temperate, and tropical forests fall below these ranges: 94% (*n* = 31/33), 77% (*n* = 103/133), and 66% (*n* = 55/83), respectively (**Figure 1B**). Thus, to date, stemflow in most forests appears to act as a water resource limitation to near-stem soils, unless subsidized by near-stem throughfall.

## Obstacles to Stemflow Infiltration Further Limit Water Supply to Near-Stem Soils

Stemflow is mostly measured by installing collars to capture stem drainage at, or near, breast height: 1.4 m above the surface—see photographs by Levia and Germer (2015). As a result, prior to entering the soil, stemflow must first pass through the various covers at the stem base and forest floor – all of which can store substantial amounts of water. Vascular and non-vascular epiphytic vegetation attach to stem bark surfaces and are ubiquitous across forest ecosystems (Zotz and Bader, 2009). Although the global distribution of cryptogamic covers is poorly understood, they inhabit all studied forest ecosystems (Elbert et al., 2012). Lichens commonly fix themselves to stem bark and can become dense near the stem base, where, for example, over one-third of lichen species have been found on the bottom 2 m of the tree stem (Marmor et al., 2013). Bryophytes (mosses, liverworts, and hornworts) are also commonly found at the stem bottom. Should the stem lack epiphytes, stemflow will still likely encounter lichen, bryophyte, or vascular vegetation in the soils immediately surrounding the trunk. The significant water storage capacity of these vegetation covers (up to 1000% of dry weight: Van Stan and Pypker, 2015), when present, can further diminish stemflow water supply to soils. When on- and near-stem vegetation cover is absent, there will be an absorbent litter layer capable of storing more water than the ≤2% of rainfall that is drained as stemflow by most forests (1–3 mm storm^-1^: Gerrits and Savenije, 2011). Gerrits and Savenije (2011) also found that forests generally lose 10–60% of net rainfall when both evaporation and storage of rainfall by the forest floor are considered. The amount of rainfall lost to the forest floor is therefore often an order of magnitude larger than that provided by stemflow generated from ≤2% of rainfall.

Volumetrically small stemflow in most forests is unlikely to bypass major water storage reservoirs in the forest floor (as has been observed for prodigious stemflow producers: Schwärzel et al., 2012). It also unlikely that rainwater traveling down the stem is sheltered significantly from evaporative drivers, as recent work found stem evaporative losses can be as high as those at the canopy edge (Van Stan et al., 2017a). These hydrometeorological conditions and water storage demands of elements at the stem base and forest floor, in conjunction with inefficient stemflow generation, can require substantial rainfall to initiate stemflow generation in most forests. This equates to fewer storms contributing stemflow to near-stem soils. Ultimately, the near-stem soils of most forests will almost certainly access only a fraction of the rainfall drained as stemflow. Even if near-stem soils receive water from lateral flow or superficial runoff, which can occur for large storms in humid forests, they still must compensate for a persistent and profound shortage of stemflow supply. Substantial throughfall has been observed near stems of significant stemflow generating forests (Bialkowski and Buttle, 2015), but the few studies on throughfall contributions near low stemflow generating stems find negligible throughfall near the stem (Guswa and Spence, 2012; Fathizadeh et al., 2014) and, although there is no consensus on throughfall pattern controls (Levia and Frost, 2006), many studies find throughfall decreases with increasing proximity to the stem—particularly in low stemflow generating stands (e.g., Johnson, 1990; Beier et al., 1993; Whelan and Anderson, 1996; Ito et al., 2008). Increased branch biomass and bark surfaces near the stem, due to major branch confluences, enhance the likelihood that throughfall is a negligible near-stem subsidy in low-stemflow generating stands due to the substantial water storage not often satisfied by negligible stem drainage (Levia and Herwitz, 2005). Whether and to what extent throughfall can supplement reduced stemflow inputs is a current knowledge gap. However, this is a stark contrast to the preferential moisture recharge (Durocher, 1990), bypass flow along roots (Martinez-Meza and Whitford, 1996), and infiltration-excess overland flow (Herwitz, 1986) that has long been associated with stemflow.

## When Stemflow Production is Low, Solute Concentrations are High

**FIGURE 2:**
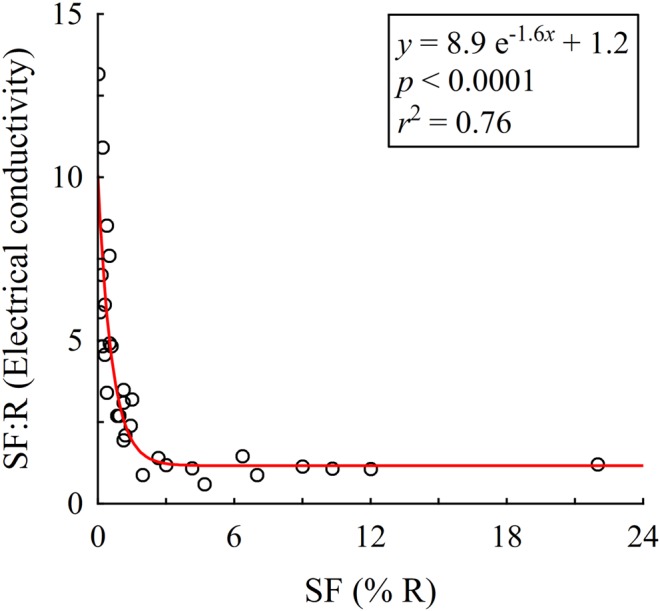
The ratio of stemflow (SF) to rainfall (R) electrical conductivity for selected studies that represent a wide range of SF values as a percentage of rainfall amount. Studies associated with each data point are provided in Supplementary Table S3.

Stemflow is arguably the longest path a rain drop can travel to reach the soil surface, requiring lengthy interaction between rainfall and canopy (mostly bark) surfaces. As a result of the many mechanisms that can exchange solutes and particulates between the canopy and rainfall, stemflow can potentially be more chemically enriched than most flow paths through the canopy (Levia and Germer, 2015). The canopy solute reservoir available to stemflow, however, is limited and intrastorm observations of efficient stemflow generators reveal that solute concentrations drop precipitously after several liters of stemflow over 0.5–1 h (Levia et al., 2011). No studies known to the authors have reported on intrastorm stemflow solute concentration dynamics for tree species that divert ≤2% of rainfall to their stems. Nevertheless, it is unlikely that low stemflow generators will deplete the canopy solute reservoir (e.g., Bade et al., 2015). In fact, stemflow dissolved organic carbon concentrations from *Quercus virginiana* (<0.2% stemflow) did not dilute with greater rainfall; rather, concentrations linearly increased with increasing storm size up to tropical storm magnitudes and intensities (Van Stan et al., 2017b). Total stemflow production from <1% of rainfall over several months across *Pinus resinosa* canopies, for example, was over six times more concentrated in Na^+^ than voluminous stemflow (nearly 6% of rainfall) from *Acer rubrum* (Mahendrappa, 1974). In fact, a selection of 31 studies spanning the full range of long-term (several months to annual mean) stemflow percentages shows that the ratio of total dissolved solids (per electrical conductivity) between stemflow and rainfall exponentially decay from 13 times greater than open rainfall (*Picea abies*: Mosello et al., 2002) to a ∼1:1 ratio when >2% of rainfall drains to the stem (**Figure 2**). An outlier study not shown in **Figure 2**, McColl (1970) found the electrical conductivity in stemflow from a Costa Rican *Inga* species (<1% of rainfall) was over 100 times greater than open rainfall. Since most studied forests generate very low stemflow volumes (**Figure 1**), the paltry water supply to soils near most tree stems may have several to 100 times the solute concentrations of open precipitation (**Figure 2**). Theoretical and observational work indicates that a combination of stemflow-induced drought conditions alongside the osmotic stresses caused by infrequent, but highly enriched, water supplies may alter microbial community structure and its traits regulating drought responses (Yuan et al., 2007; Manzoni et al., 2014).

## Stemflow may be Well-Seasoned, but it is a Meager Meal

Well-researched generous stemflow producers, like *Fagus sylvatica* in Europe (a Web of Science search for this species, “stemflow,” and “Europe” returned > 80 studies), despite their low solute concentrations, <5 mg l^-1^ (Staelens, 2006), produce large solute fluxes that can concentrate up to 20% of per ha fluxes for some nutrients (i.e., K^+^ and Mg^2+^) to 10–50 m^2^ ha^-1^ of basal area, depending on the stand (Berger et al., 2009). In contrast, *Larix* species that inhabit three continents’ boreal forests generate <1% of stemflow over 20–40 m^2^ ha^-1^ of basal area, depending on the stand (Ovington, 1954; Cape et al., 1991; Toba and Ohta, 2005), yet a Web of Science search for the entire genus “*Larix*” and “stemflow” returns only 25 publications. *Larix* and the many other inefficient stemflow-generating genera globally—*Abies* (Negi et al., 1998), *Pinus* (Santa Regina and Tarazona, 2000), *Quercus* (Moreno et al., 2001), *Tsuga* (Ovington, 1954) etc.—can limit soil nutrient flux just as impressively as efficient stemflow generators can augment it. Stemflow from *Picea* species in the subarctic, for example, supplied <4 and <1% of the annual rainfall K^+^ and NO_3_^-^ flux, respectively, to near-stem soils (Moore, 1980). In multiple tropical rainforest sites, total cation and anion solute fluxes from stemflow to near-stem soils were also low, 0.1–0.6 g m^-2^ y^-1^ (Tobón et al., 2004). Stemflow in a subtropical *Lithocarpus–Castenopsis* forest supplied <0.1 g-N m^-2^ y^-1^ and <0.01 g-P m^-2^ y^-1^ to near-stem soils—an order of magnitude less than precipitation flux despite the removal of epiphytes (Liu et al., 2002). Near-stem soils in temperate hardwood stands containing voluminous stemflow producers, like the aforementioned *Fagus sylvatica*, alongside low stemflow generators (*Fraxinus excelsior*, *Carpinus betulus*, and others) can receive strikingly low solute fluxes at the stand scale: being just 3.7% of throughfall flux (Talkner et al., 2010). Thus, soils near low stemflow generating trees may not only experience severe water limitations (**Figure 1**) and elevated stemflow solute concentrations (**Figure 2**), but limited solute flux.

Low stemflow conditions may affect the soil environment and its biogeochemical processes, both individually and in combination. For example, rainfall reductions to forest soils alone have been linked to increased methane uptake (Fest et al., 2017) and shifts in drying-rewetting regimes can alter soil aggregation and aggregate stability (Kaiser et al., 2015). Coupled water-solute flux limitation has been linked to shifts in soil microbial community composition (Rosier et al., 2015) and N-functioning genes (Moore et al., 2016). Limited soil environments, especially those occasionally doused with salty precipitation, also tend to select for lichens and bryophytes due to their high tolerances for desiccation and poor nutrient availability (Proctor et al., 2007; Kranner et al., 2008). These example impacts on the soil environment contrast with those observed and hypothesized for soils receiving substantial stemflow (see discussion of impacts in Introduction).

## Concluding Remarks and Recommended Future Directions

This mini-review finds that, for most soils near tree stems, the stemflow story is not one of hydrological abundance and the associated biogeochemical processes often described in reviews. Soils near the stems of inefficient stemflow generators may experience little water and nutrient supply and, when stemflow does arrive, it can be 10–100 times more chemically enriched than rainfall or throughfall. This has implications for near-stem soil ecological processes, yet very few studies known to the authors have investigated the effects of stemflow limitation on edaphic conditions and functions (Koch and Matzner, 1993). Stemflow limitation in forests, therefore, merits the attention of scientists working at the intersection of plant ecological and hydrological processes. Questions on this topic abound, including: can throughfall mitigate stemflow-related resource limitations? How do drying-rewetting cycles and near-stem edaphic conditions compare between soils beside high and low stemflow generators? Are near-stem soil microbial communities and/or their functions influenced by low stemflow generation? Does stemflow scarcity versus abundance vary with stand age or management?

## Data Accessibility

All data are available in the supporting information or cited publications.

## Author Contributions

JVS conceived the study and supervised the literature synthesis and analysis. DG assembled data from the literature and assisted in data analysis. Both authors contributed critically to manuscript preparation.

## Conflict of Interest Statement

The authors declare that the research was conducted in the absence of any commercial or financial relationships that could be construed as a potential conflict of interest.
