# Supplementary material for: Mini-Review: Stemflow as a Resource Limitation to Near-Stem Soils
Source: Front Plant Sci. 2018 Feb 27;9:248. doi: 10.3389/fpls.2018.00248 (PMC5835114; doi:10.3389/fpls.2018.00248)
Supplement: Supplementary file 1 [file Data_Sheet_1.docx]

**Supplementary Material**

Van Stan, J.T. and Gordon, D.A., Mini-Review: Stemflow as a Resource Limitation to Near-Stem Soils

**Table S1**. Latitude and longitude coordinates for studies reporting that stemflow was measured (used to generate map shown in Figure 1).

| Latitude | Longitude | Study |  |
| --- | --- | --- | --- |
| 28.3 | -16.6 | Aboal et al. (2002) | |
| 60.4 | 9.7 | Abrahamsen et al. (1977) | |
| 53.1 | -8.4 | Aherne et al. (1999) | |
| 16 | -15 | Akpo et al. (2005) | |
| 42.5 | -0.7 | Alvera (1976) | |
| 55.3 | -2.6 | Anderson and Pyatt (1986) | |
| 59 | 12.3 | Andersson (1991) | |
| -1.3 | 112.4 | Asdak et al. (1998) | |
| 48.7 | 6.2 | Aussenac (1968) | |
| 27.5 | 88.5 | Awasthi et al. (1995) | |
| 31.3 | -7.7 | Badri and Gauquelin (2003) | |
| 52.3 | -115.3 | Baker et al. (1977) | |
| 57 | -112.1 | Baker et al. (1977) | |
| 51.4 | 30.1 | Belli et al. (1996) | |
| 56 | 13.5 | Bergkvist and Folkeson, (1992) | |
| 51 | 10 | Bittner et al. (2010) | |
| 19.4 | -155.6 | Brauman et al. (2010) | |
| 15.2 | -89.7 | Brown et al. (1996) | |
| 32.4 | -84.8 | Bryant et al. (2005) | |
| 40.9 | -5.2 | Calabuig et al. (1978) | |
| 54 | -2.4 | Cape et al. (1991) | |
| 57.1 | -3.1 | Cape et al. (1991) | |
| 54.4 | -3 | Carlisle et al. (1967) | |
| 8.7 | -82.2 | Cavelier et al. (1997) | |
| -40.8 | -63 | Cecchi et al. (2006) | |
| 49.9 | 10.5 | Chang and Matzner (2000) | |
| 5 | 117.8 | Chappell et al. (2001) | |
| 12.9 | 102.3 | Chunpaga and Wachirajutipong (1977) | |
| 5.3 | 9.1 | Chuyong et al. (2004) | |
| -33.7 | 23.6 | Cowling and Mills (2011) | |
| -35 | 145 | Crockford and Richardson (1990) | |
| 38.5 | -8 | David et al. (2006) | |
| 51 | 8.2 | Delfs (1967) | |
| 36.9 | -93 | DeWalle and Paulsell (1979) | |
| 5.5 | -62 | Dezzeo and Chacon (2006) | |
| 44.4 | 3.7 | Didon-Lescot (1996) | |
| 50.9 | 20.6 | Dolegowska and Migaszewski (2013) | |
| 52.6 | 4.7 | Dolman (1987) | |
| 36.8 | -2.5 | Domingo et al. (1994) | |
| -28.6 | 32.1 | Dovey et al. (2011) | |
| -35.6 | 150.3 | Dunin et al. (1988) | |
| 51.5 | -2.6 | Durocher (1990) | |
| 4.5 | 115.2 | Dykes (1997) | |
| -4.1 | 137.2 | Edwards (1982) | |
| 44.3 | -100 | Ellsbury et al. (1996) | |
| 36.8 | 34.6 | Eren and Hatipoglu-Bagci (2010) | |
| 26.6 | 118.1 | Fan and Hong (2001) | |
| 40.6 | -8.5 | Ferreira (1992) | |
| -4 | -79.1 | Fleischbein et al. (2006) | |
| 54.7 | -83.2 | Foster (1974) | |
| -54.1 | -68 | Frangi et al. (2005) | |
| 44.9 | -63.7 | Freedman and Prager (1986) | |
| 14.1 | 121.2 | Gascon (1994) | |
| 52.4 | 0.7 | Gash and Morton (1978) | |
| -14.8 | -62.4 | Gerold et al. (2008) | |
| 27.6 | 85.6 | Ghimire et al. (2012) | |
| -18.9 | 48.4 | Ghimire et al. (2017) | |
| 44 | 11 | Giacomin and Trucchi (1992) | |
| 68.7 | -134.1 | Gill (1975) | |
| -41 | -72 | Godoy et al. (1999) | |
| 37.9 | -4.8 | Gomez et al. (2002) | |
| -10.5 | -75.3 | Gomez-Peralta et al. (2008) | |
| 47.5 | 19 | Gonczol and Revay (2004) | |
| 46.9 | 26.3 | Gonczol and Revay (2004) | |
| 59.4 | 18 | Gonczol and Revay (2004) | |
| 40.9 | -4 | Gonzalez-Cascon et al. (1994) | |
| 24.6 | 93.8 | Gupta and Usharani (2009) | |
| 35 | 50.4 | Hakimi et al. (2017) | |
| 7.2 | 80.7 | Hall et al. (1996) | |
| 48.6 | -115.1 | Hauck and Spribille (2005) | |
| 35.3 | -83.9 | Helvey (1967) | |
| -17.3 | 145.9 | Herwitz (1986) | |
| 17.1 | 54 | Hildebrandt and Eltahir (2006) | |
| 9.6 | -83.8 | Holscher et al. (2003) | |
| 48.1 | 16.7 | Hoppe (1896) | |
| 5.8 | -7.1 | Hutjes et al. (1990) | |
| 43.6 | 4.2 | Ibrahim et al. (1982), Rapp and Ibrahim (1978) | |
| 36 | 140 | Iida et al. (2005) | |
| 39.3 | 16.5 | Iovino et al. (1998) | |
| 45.1 | 7.8 | Iovino et al. (1998) | |
| -4.8 | 38.5 | Jackson (1971) | |
| 10.4 | 77 | James and Pradeepkumar (1987) | |
| 5.3 | -59.4 | Jetten (1996) | |
| 56 | -4.3 | Johnson (1990) | |
| 58.7 | 26.75 | Kabral and Frey (2009), “Saarejärve” | |
| 58.3 | 21.9 | Kabral and Frey (2009), “Vilsandi” | |
| -38.6 | 176.4 | Kelliher et al. (1992) | |
| 19.5 | -96.5 | Kellman and Roulet (1990) | |
| 20 | 73.1 | Khan (1991) | |
| -43.2 | 146.3 | Kirkpatrick (1997) | |
| 49.3 | 19.3 | Kluciarova et al. (2008) | |
| 51.1 | 10.4 | Kramer and Holscher (2009) | |
| 50.8 | 15.3 | Krecek et al. (2017) | |
| 23.2 | 71.5 | Krishna et al. (2012) | |
| -4 | 12 | Laclau et al. (2003) | |
| 34.5 | -94.5 | Lawson (1967) | |
| 37.5 | 127.5 | Lee et al. (1997) | |
| 33.4 | 99.4 | Lei et al. (1994) | |
| 47.1 | 128.8 | Lei et al. (1994) | |
| 27.4 | -80.4 | Li et al. (1997a) | |
| 36.2 | 103.8 | Li et al. (2008) | |
| -18.9 | -48.3 | Lilienfein and Wilcke (2004) | |
| 23.9 | 120.8 | Liu and Sheu (1999) | |
| 31.6 | 102.7 | Liu et al. (2001) | |
| 24.5 | 101 | Liu et al. (2002) | |
| 21.8 | 101.2 | Liu et al. (2008) | |
| 42.2 | 1.8 | Llorens et al. (1997) | |
| -3 | -60 | Lloyd and Marques (1988) | |
| 34.2 | -117.8 | Lockwood (1985) | |
| 37.2 | -119.5 | Lockwood (1985) | |
| 38.8 | -121.2 | Lockwood (1985) | |
| 29.7 | 94.4 | Lu et al. (2016) | |
| 13.8 | 100.5 | Luangjame et al. (2001) | |
| 14.7 | 101.3 | Luangjame et al. (2001) | |
| 16.1 | 103.6 | Luangjame et al. (2001) | |
| 29.6 | -82.4 | Lugo et al. (1976) | |
| 62.3 | 58.9 | Lukina (2007), “Pechoro-Ilych Reserve” | |
| 64.6 | 31.9 | Lukina (2008), “Karelia Site” | |
| 67.9 | 31.9 | Lukina (2008), “Lapland State Nature Reserve” | |
| 55.8 | 36 | Luschev (1940), “Istra Forest” | |
| 55 | 39.5 | Luschev (1940), “Oka River Forest” | |
| 53.9 | 36.9 | Luschev (1940), “Prokudin Forest” | |
| 56 | 38 | Luschev (1940), “Pushkino Forest” | |
| 45.3 | -68.3 | Mahendrappa and Ogden(1973) | |
| 21.5 | -158.1 | Mair and Fares (2010) | |
| 43.7 | -80 | Malone (2015) | |
| 4.2 | 114 | Manfroi et al. (2006) | |
| 3 | 102.3 | Manokaran (1979) | |
| -1.4 | -70.7 | Marin et al. (2000) | |
| -0.4 | -71.6 | Marin et al. (2000) | |
| 0.6 | -72.4 | Marin et al. (2000) | |
| 39.5 | -6.4 | Mateos and Schnabel (1998) | |
| 31.8 | -106.5 | Mauchamp and Janeau (1993) | |
| 41.1 | 20.9 | Melovski et al. (1992) | |
| 40 | 20.8 | Michopoulos et al. (2001) | |
| 12.8 | 123.3 | Miole et al. (2011) | |
| 64.5 | 40.6 | Molchanov (1960), “Arkhangelsk Forest” | |
| 51.4 | 42 | Molchanov (1960), “Tellerman Experimental Forest” | |
| 55 | -66.9 | Moore (1980) | |
| 56.8 | 97.5 | Moore (2003) | |
| 40.2 | -6.6 | Moreno et al. (1993) | |
| 40.1 | -3 | Moreno et al. (2001) | |
| 46.1 | 12.4 | Mosello et al. ( 2002) | |
| 42.2 | 13.7 | Mosello et al. (2002) | |
| 43.5 | 10.5 | Mosello et al. (2002) | |
| 44.6 | 9.2 | Mosello et al. (2002) | |
| -7.1 | 37.6 | Munishi and Shear (2005) | |
| 24.8 | -99.6 | Navar and Bryan (1990) | |
| 51.2 | -1.2 | Neal et al. (1993) | |
| 45.2 | -78.8 | Neary and Gizyn (1994) | |
| 30.1 | 80 | Negi et al. (1998) | |
| 56.1 | 13.4 | Nihlgard (1970) | |
| 48.4 | 2.6 | Nizinski and Saugier (1989) | |
| 49.8 | 5.7 | Noirfalise (1959) | |
| -32.1 | 116.4 | Nulsen et al. (1986) | |
| 7.4 | 4 | Opakunle (1989) | |
| 44 | -103.8 | Orr (1972) | |
| 51.1 | 0.4 | Ovington (1954) | |
| 29.4 | -99.7 | Owens et al. (2006) | |
| -40.8 | -72.2 | Oyarzun et al. (2004) | |
| 29.5 | 79.5 | Pathak et al. (1985) | |
| 58 | -134.4 | Patric (1966) | |
| 50.6 | 6.1 | Petit and Kamutanda (1984) | |
| 63.9 | 28.5 | Piirainen et al. (1998) | |
| 42.7 | 25 | Pipkov et al. (2000) | |
| 47.3 | -71.1 | Plamondon et al. (1984) | |
| 41.8 | -7.3 | Portela and Pires (1995) | |
| -25.7 | 150.1 | Prebble and Stirk (1980) | |
| -21 | 142.7 | Pressland (1973) | |
| 45.6 | -79.7 | Price and Carlyle-Moses (2003) | |
| 55.5 | -98.2 | Price et al. (1997) | |
| -0.6 | -90.4 | Pryet et al. (2012) | |
| 39.3 | -86.4 | Pryor and Barthelmie (2005) | |
| 40 | 115.4 | Qiang and Zhi (2000) | |
| 3.1 | 101.7 | Radzi Abas (1992) | |
| 39.4 | -123.7 | Reid and Lewis (2009) | |
| 41.8 | 2.4 | Rodrigo et al. (2003) | |
| 41.2 | -77.2 | Rogerson and Byrnes (1968) | |
| 44.2 | -122.2 | Rothacher (1963) | |
| -21.1 | 14.3 | Roth-Nebelsick et al. (2012) | |
| 36.5 | 51.5 | Sadeghi et al. (2017) | |
| 42.9 | -114 | Saffigna et al. (1976) | |
| 16.1 | -61.67 | Sansoulet et al. (2007) | |
| 42.3 | -3.1 | Santa Regina and Tarazona (2000) | |
| 50.9 | -120.4 | Schooling and Carlyle-Moses (2015) | |
| -3.1 | -59.9 | Schroth et al. (2001) | |
| 38.1 | -1.5 | Serrato and Diaz (1998) | |
| 24.7 | -99.9 | Silva and Rodrigues (2001) | |
| 32.7 | 35 | Singer et al. (1996) | |
| 31.1 | 77.2 | Singh (1987) | |
| 6 | 116.1 | Sinun et al. (1992) | |
| 34.7 | -111.7 | Skau (1964) | |
| -23 | 133 | Slayter (1965) | |
| -38.2 | 147.4 | Specht (1957) | |
| 48.9 | -125 | Spittlehouse (1998) | |
| 12.8 | 74.9 | Sridhar and Karamchand (2009) | |
| 24.5 | 83 | Srivastava and Ambasht (1994) | |
| 51 | 3.8 | Staelens (2006) | |
| 6.4 | -66.6 | Steinhardt (1979) | |
| 16.9 | 101.1 | Takahashi et al. (1983) | |
| 18.8 | 98.9 | Tanaka et al. (2005) | |
| 3 | 102.3 | Tani et al. (2003) | |
| 30 | -100 | Thurow et al. (1987) | |
| 26.8 | 109.7 | Tian et al. (1994) | |
| 35.2 | 137 | Toba and Ohta (2005) | |
| 39.7 | 140.9 | Toba and Ohta (2005) | |
| 62.2 | 129.6 | Toba and Ohta (2005) | |
| 42.5 | 23 | Tsakov and Alexandrov (2005) | |
| -4.6 | 143 | Turvey (1974) | |
| 26 | -81.7 | Twilley and Chen (1998) | |
| -10 | -61.7 | Ubarana (1996) | |
| -39.7 | -73.2 | Uyttendaele and Iroumé ( 2002) | |
| 38.6 | -8.6 | Valente et al. (1997) | |
| 38.8 | -8.9 | Valente et al. (1997) | |
| 64.9 | -147.7 | Van Cleve et al. (1983), Viereck et al. (1983) | |
| 39.7 | -75.9 | Van Stan (2012) | |
| 32 | -81 | Van Stan et al. (2017) | |
| 4.8 | -75.5 | Veneklaas and Van Ek (1990) | |
| -0.1 | 114 | Vernimmen et al. (2007) | |
| 47.5 | -93.5 | Verry and Timmons (1977) | |
| 48.2 | 7.2 | Viville et al. (1993) | |
| 41.6 | 73.1 | Voigt (1960) | |
| 18.4 | -65.7 | Weaver (1972) | |
| 41.9 | -112.7 | West and Gifford (1976) | |
| 56.2 | -4.6 | Wheater et al. (1987) | |
| 1.4 | 173 | White et al. (2007) | |
| 32.3 | -106.7 | Whitford et al. (1997) | |
| -9.1 | 125.1 | Widiyono (2010) | |
| 49.6 | -114.5 | Williams et al. (2012) | |
| 39.8 | -105.9 | Wilm and Niederhof (1941) | |
| 12.7 | 101.4 | Witthawatchutikui and Suksawang (1987) | |
| 27.8 | 114.6 | Xiaoi et al. (2007) | |
| 23.2 | 112.5 | Yan et al. (2003) | |
| 41 | -121 | Young et al. (1984) | |
| 46 | 14.5 | Zabret and Sraj (2015) | |
| 18.7 | 108.8 | Zeng (1994) | |
| 11.2 | 125 | Zhang et al. (2015) | |
| 35.5 | 106.3 | Zhongjie et al. (2010) | |
| 50.8 | 121.5 | Zhou (2003) | |

**Table S2**. Stemflow as a percentage of rainfall across the canopy area for all studies selected for the minireview (values used to generate the histograms in Figure 1).

| Climate | Stemflow:Rainfall | Study |
| --- | --- | --- |
| Mediterranean | 6.9 | Aboal et al.(2002) |
| Boreal | 2.3 | Abrahamsen et al. (1977) |
| Boreal | 0.1 | Abrahamsen et al. (1977) |
| Boreal | 1.1 | Abrahamsen et al. (1977) |
| Temperate | 1.1 | Aherne et al. (1999) |
| Tropical | 2.8 | Akpo et al. (2005) |
| Mediterranean | 0.8 | Alvera (1976) |
| Temperate | 1.0 | Anderson and Pyatt (1986) |
| Boreal | <1 | Andersson (1991) |
| Tropical | 0.4 | Asdak et al. (1998) |
| Tropical | 1.4 | Asdak et al. (1998) |
| Temperate | 0.8 | Aussenac (1968) |
| Temperate | 1.0 | Aussenac (1968) |
| Temperate | 1.0 | Aussenac (1968) |
| Mediterranean | 2.1 | Badri and Gauquelin (2003) |
| Temperate | 0.4 | Bahmani et al. (2012) |
| Boreal | 2.5 | Bergkvist and Folkeson (1995) |
| Temperate | 4.0 | Bittner et al. (2010) |
| Tropical | <1 | Brauman et al. (2010) |
| Tropical | 1.8 | Brown et al. (1996) |
| Subtropical | 0.5 | Bryant et al. (2005) |
| Subtropical | 0.5 | Bryant et al. (2005) |
| Subtropical | 0.5 | Bryant et al. (2005) |
| Subtropical | 1.9 | Bryant et al. (2005) |
| Subtropical | 0.7 | Bryant et al. (2005) |
| Mediterranean | 0.6 | Calabuig et al. (1978) |
| Temperate | 9.0 | Cape et al. (1991) |
| Boreal | 0.7 | Cape et al. (1991) |
| Temperate | 13.5 | Cape et al. (1991) |
| Temperate | 6.5 | Cape et al. (1991) |
| Temperate | 10.0 | Cape et al. (1991) |
| Temperate | 2.2 | Carlisle et al. (1967) |
| Tropical | 0.4 | Cavelier et al. (1997) |
| Temperate | 3.6 | Cecchi et al. (2006) |
| Temperate | 6.6 | Chang and Matzner (2000) |
| Tropical | 1.0 | Chappell et al. (2001) |
| Tropical | 2.0 | Charoensuk et al. (1989) |
| Tropical | 0.0 | Chunkao et al. (1971) |
| Tropical | 0.0 | Chunkao et al. (1971) |
| Tropical | 1.0 | Chunkao et al. (1971) |
| Tropical | 0.2 | Chunpaga and Wachirajutipong (1977) |
| Tropical | 1.5 | Chuyong et al. (2004) |
| Tropical | 2.2 | Chuyong et al. (2004) |
| Boreal | 0.7 | Courchesne and Hendershot (1988) |
| Boreal | 2.2 | Courchesne and Hendershot (1988) |
| Boreal | 2.7 | Courchesne and Hendershot (1988) |
| Boreal | 0.3 | Courchesne and Hendershot (1988) |
| Subtropical | <1 | Cowling and Mills (2011) |
| Temperate | 4.1 | Crockford and Richardson (1990) |
| Temperate | 8.9 | Crockford and Richardson (1990) |
| Mediterranean | 0.3 | David et al. (2006) |
| Temperate | 0.8 | Delfs (1967) |
| Tropical | 1.6 | Dezzeo and Chacon (2006) |
| Tropical | 6.8 | Dezzeo and Chacon (2006) |
| Tropical | 8.4 | Dezzeo and Chacon (2006) |
| Mediterranean | 0.7 | Didon-Lescot (1996) |
| Temperate | 2.5 | Dolman (1987) |
| Mediterranean | 1.5 | Domingo et al. (1994) |
| Subtropical | 5.0 | Dovey et al. (2011) |
| Subtropical | 2.0 | Dovey et al. (2011) |
| Subtropical | 2.9 | Dunin et al. (1988) |
| Temperate | 3.8 | Durocher (1990) |
| Tropical | 1.0 | Dykes (1997) |
| Tropical | 0.8 | Edwards (1982) |
| Subtropical | 3.0 | Fan and Hong (2001) |
| Desert | <1 | Fathizadeh et al. (2013) |
| Mediterranean | 2.9 | Ferreira (1992) |
| Mediterranean | 1.1 | Ferreira (1992) |
| Tropical | 1.0 | Fleischbein et al. (2006) |
| Tropical | 1.1 | Fleischbein et al. (2006) |
| Tropical | 0.9 | Fleischbein et al. (2006) |
| Boreal | 2.0 | Foster (1974) |
| Temperate | 0.1 | Frangi et al. (2005) |
| Boreal | 0.2 | Freedman and Prager (1986) |
| Boreal | 1.0 | Freedman and Prager (1986) |
| Tropical | 2.2 | Gascon (1994) |
| Tropical | 1.1 | Gascon (1994) |
| Temperate | 1.9 | Gash and Morton (1978) |
| Tropical | 0.8 | Gerold et al. (2008) |
| Subtropical | 1.4 | Ghimire et al. (2012) |
| Subtropical | 0.5 | Ghimire et al. (2012) |
| Tropical | 1.7 | Ghimire et al. (2012) |
| Temperate | 0.3 | Ghorbani and Rahmani (2009) |
| Mediterranean | 13.6 | Giacomin and Trucchi (1992) |
| Subarctic | <1 | Gill (1975) |
| Temperate | 9.0 | Godoy et al. (1999) |
| Mediterranean | 3.8 | Gomez et al. (2002) |
| Tropical | 0.2 | Gomez-Peralta et al. (2008) |
| Tropical | 0.2 | Gomez-Peralta et al. (2008) |
| Mediterranean | 0.3 | Gonzalez-Cascon et al. (1994) |
| Subtropical | 2.5 | Gupta and Usharani (2009) |
| Temperate | 2.0 | Helvey (1967) |
| Tropical | 13.6 | Herwitz (1986) |
| Desert | 30.0 | Hildebrandt and Eltahir (2006) |
| Subtropical | 2.2 | Holscher et al. (2003) |
| Temperate | 0.1 | Hoppe (1896) |
| Temperate | 0.7 | Hoppe (1896) |
| Temperate | 0.3 | Hosseini et al. (2011) |
| Tropical | 0.3 | Hutjes et al. (1990) |
| Mediterranean | 2.3 | Ibrahim et al. (1982) |
| Temperate | 1.0 | Pipkov et al. (2000) |
| Temperate | 1.2 | Iida et al. (2005) |
| Temperate | 0.5 | Iida et al. (2005) |
| Mediterranean | 0.8 | Iovino et al. (1998) |
| Mediterranean | 0.3 | Iovino et al. (1998) |
| Tropical | 0.9 | Jackson (1971) |
| Tropical | 4.0 | James and Pradeepkumar (1987) |
| Tropical | 0.9 | Jetten (1996) |
| Tropical | 1.5 | Jirasuktaveekul et al. (1987) |
| Temperate | 3.0 | Johnson (1990) |
| Mediterranean | 4.0 | Kelliher et al. (1992) |
| Mediterranean | 7.0 | Kelliher et al. (1992) |
| Tropical | 0.6 | Kellman and Roulet (1990) |
| Desert | 0.7 | Khan (1991) |
| Desert | 1.3 | Khan (1991) |
| Temperate | 2.5 | Kramer and Holscher (2009) |
| Temperate | <1 | Krecek et al. (2017) |
| Subtropical | 1.6 | Laclau et al. (2003) |
| Temperate | 2.0 | Lawson (1967) |
| Temperate | 1.0 | Lazerjan (2012) |
| Subtropical | 1.1 | Lee et al. (1997) |
| Subtropical | 1.1 | Lee et al. (1997) |
| Temperate | 2.6 | Lei et al. (1994) |
| Temperate | 5.0 | Lei et al. (1994) |
| Temperate | 2.3 | Lei et al. (1994) |
| Subtropical | 1.0 | Li et al. (1997a) |
| Subtropical | 0.5 | Li et al. (1997a) |
| Subtropical | 0.8 | Lilienfein and Wilcke (2004) |
| Subtropical | 0.8 | Lilienfein and Wilcke (2004) |
| Subtropical | 3.0 | Liu and Sheu (1999) |
| Temperate | <1 | Liu et al. (2001) |
| Subtropical | 2.0 | Liu et al. (2002) |
| Temperate | 2.0 | Liu et al. (2003) |
| Subtropical | 8.8 | Liu et al. (2008) |
| Subtropical | 6.2 | Liu et al. (2008) |
| Desert | 1.7 | Llorens and Domingo (2007), "S03" Site |
| Mediterranean | 1.3 | Llorens et al. (1997) |
| Tropical | 1.8 | Lloyd and Marques (1988) |
| Tropical | 1.8 | Lloyd et al. (1988) |
| Tropical | 1.3 | Lu et al. (2016) |
| Subtropical | 4.8 | Lugo et al. (1976) |
| Boreal | 0.0 | Luschev (1940) |
| Boreal | 0.1 | Luschev (1940) |
| Temperate | 1.0 | Mahendrappa and Ogden (1973) |
| Temperate | 5.6 | Mahendrappa (1974) |
| Temperate | 1.6 | Mahendrappa (1974) |
| Temperate | 2.3 | Mahendrappa (1974) |
| Temperate | 0.7 | Mahendrappa (1974) |
| Temperate | 5.3 | Mahendrappa (1974) |
| Temperate | 6.1 | Mahendrappa (1974) |
| Tropical | 33.9 | Mair and Fares (2010) |
| Tropical | 3.6 | Mair and Fares (2010) |
| Temperate | 2.2 | Majima and Tase (1982) |
| Temperate | 0.6 | Malone (2015) |
| Temperate | 0.8 | Malone (2015) |
| Tropical | 3.1 | Manfroi et al. (2006) |
| Tropical | 0.6 | Manokaran (1979) |
| Tropical | 1.1 | Marin et al. (2000) |
| Tropical | 0.9 | Marin et al. (2000) |
| Tropical | 1.5 | Marin et al. (2000) |
| Tropical | 0.9 | Marin et al. (2000) |
| Mediterranean | 0.7 | Mateos and Schnabel (1998) |
| Mediterranean | 8.0 | Michopoulos et al. (2001) |
| Tropical | 1.0 | Miole et al. (2011) |
| Boreal | 3.4 | Molchanov (1960) |
| Boreal | 0.2 | Molchanov (1960) |
| Boreal | 4.0 | Molchanov (1960) |
| Boreal | 0.4 | Molchanov (1960) |
| Subarctic | 0.1 | Moore (1980) |
| Boreal | 0.4 | Moore (2003) |
| Boreal | 0.9 | Moore (2003) |
| Boreal | 3.5 | Moore (2003) |
| Mediterranean | 0.9 | Moreno et al. (2001) |
| Mediterranean | 0.7 | Moreno et al. (2001) |
| Mediterranean | 0.6 | Moreno et al. (2001) |
| Mediterranean | 0.8 | Moreno et al. (2001) |
| Mediterranean | 1.1 | Mosello et al. (2002) |
| Mediterranean | 4.1 | Mosello et al. (2002) |
| Mediterranean | 2.0 | Mosello et al. (2002) |
| Mediterranean | 3.1 | Mosello et al. (2002) |
| Mediterranean | 3.4 | Mosello et al. (2002) |
| Mediterranean | 4.7 | Mosello et al. (2002) |
| Tropical | 1.2 | Munishi and Shear (2005) |
| Tropical | 1.0 | Munishi and Shear (2005) |
| Temperate | 0.5 | Murai (1970) |
| Subtropical | 0.6 | Navar and Bryan (1990) |
| Subtropical | 0.8 | Navar (1993) |
| Subtropical | 1.0 | Navar (1993) |
| Temperate | 4.6 | Neal et al. (1993) |
| Temperate | 2.4 | Neary and Gizyn (1994) |
| Subtropical | 0.0 | Negi et al. (1998), Loshali and Singh (1992) |
| Subtropical | 0.2 | Negi et al. (1998), Loshali and Singh (1992) |
| Subtropical | 0.1 | Negi et al. (1998), Loshali and Singh (1992) |
| Boreal | 11.0 | Nihlgard (1970) |
| Boreal | 2.9 | Nihlgard (1970) |
| Temperate | 2.0 | Noirfalise (1959) |
| Temperate | 0.1 | Noirfalise (1959) |
| Temperate | 0.1 | Noirfalise (1959) |
| Subtropical | 5.0 | Nulsen et al. (1986) |
| Tropical | 1.4 | Onarsa (1995) |
| Tropical | 1.9 | Opankunle (1989) |
| Temperate | 0.1 | Ovington (1954) |
| Temperate | 0.1 | Ovington (1954) |
| Temperate | 0.1 | Ovington (1954) |
| Temperate | 0.1 | Ovington (1954) |
| Temperate | 0.1 | Ovington (1954) |
| Temperate | 0.2 | Ovington (1954) |
| Temperate | 0.2 | Ovington (1954) |
| Temperate | 0.1 | Ovington (1954) |
| Temperate | 0.3 | Ovington (1954) |
| Temperate | 0.3 | Ovington (1954) |
| Temperate | 0.1 | Ovington (1954) |
| Temperate | 0.1 | Ovington (1954) |
| Mediterranean | 5.0 | Owens et al. (2006) |
| Temperate | 1.4 | Oyarzun et al. (2004) |
| Subtropical | 0.4 | Pathak et al. (1985) |
| Subtropical | 0.3 | Pathak et al. (1985) |
| Subtropical | 0.2 | Pathak et al. (1985) |
| Subtropical | 0.9 | Pathak et al. (1985) |
| Subtropical | 0.9 | Pathak et al. (1985) |
| Subtropical | 0.4 | Pathak et al. (1985) |
| Boreal | <1 | Patric (1966) |
| Tropical | 1.4 | Paungchareon (1987) |
| Temperate | 3.0 | Petit and Kamutanda (1984) |
| Temperate | 0.9 | Petit and Kamutanda (1984) |
| Boreal | 1.4 | Piirainen et al. (1998) |
| Boreal | <1 | Plamondon et al. (1984) |
| Mediterranean | 0.2 | Portela and Pires (1995) |
| Subtropical | 0.8 | Prebble and Stirk (1980) |
| Temperate | 3.7 | Price and Carlyle-Moses (2003) |
| Boreal | 1.0 | Price et al. (1997) |
| Tropical | 0.6 | Pryet et al. (2012) |
| Tropical | 0.7 | Pryet et al. (2012) |
| Temperate | 2.0 | Pryor and Barthelmie (2005) |
| Temperate | 9.0 | Pryor and Barthelmie (2005) |
| Temperate | 5.0 | Pryor and Barthelmie (2005) |
| Temperate | 2.0 | Pryor and Barthelmie (2005) |
| Tropical | 2.2 | Pukjaloon et al. (1984) |
| Tropical | 1.5 | Pukjaloon et al. (1984) |
| Temperate | 1.8 | Qiang and Zhi (2000) |
| tropical | 1.2 | Radzi Abas et al. (1992) |
| Temperate | 2.5 | Reid and Lewis (2009) |
| Temperate | <1 | Reynolds and Henderson (1967) |
| Temperate | <1 | Reynolds and Henderson (1967) |
| Mediterranean | 2.7 | Rodrigo et al. (2003) |
| Temperate | 2.0 | Rogerson and Byrnes (1968) |
| Temperate | 1.0 | Rogerson and Byrnes (1968) |
| Temperate | 0.3 | Rothacher (1963) |
| Temperate | 6.0 | Sadeghi et al. (2016) |
| Temperate | 8.0 | Sadeghi et al. (2016) |
| Temperate | 2.0 | Sadeghi et al. (2016) |
| Temperate | 1.0 | Sadeghi et al. (2016) |
| Temperate | 15.0 | Sadeghi et al. (2017) |
| Subtropical | 0.8 | San Jose and Montes (1992) |
| Mediterranean | 6.5 | Santa Regina and Tarazona (2000) |
| Mediterranean | 0.5 | Santa Regina and Tarazona (2000) |
| Temperate | 1.0 | Schooling and Carlyle-Moses (2015) |
| Temperate | 0.2 | Schooling and Carlyle-Moses (2015) |
| Temperate | 1.3 | Schooling and Carlyle-Moses (2015) |
| Temperate | 0.4 | Schooling and Carlyle-Moses (2015) |
| Temperate | 0.0 | Schooling and Carlyle-Moses (2015) |
| Temperate | 0.3 | Schooling and Carlyle-Moses (2015) |
| Temperate | 0.4 | Schooling and Carlyle-Moses (2015) |
| Temperate | 1.3 | Schooling and Carlyle-Moses (2015) |
| Temperate | 2.4 | Schooling and Carlyle-Moses (2015) |
| Temperate | 0.3 | Schooling and Carlyle-Moses (2015) |
| Temperate | 0.1 | Schooling and Carlyle-Moses (2015) |
| Temperate | 1.3 | Schooling and Carlyle-Moses (2015) |
| Temperate | 2.4 | Schooling et al. (2017) |
| Temperate | 4.8 | Schooling et al. (2017) |
| Temperate | 0.2 | Schooling et al. (2017) |
| Tropical | 2.8 | Schroth et al. (2001) |
| Tropical | 0.4 | Schroth et al. (2001) |
| Tropical | 0.1 | Schroth et al. (2001) |
| Tropical | 0.7 | Schroth et al. (2001) |
| Tropical | 3.7 | Schroth et al. (2001) |
| Subtropical | 0.5 | Silva and Rodrigues (2001) |
| Subtropical | 0.6 | Silva and Rodrigues (2001) |
| Subtropical | 0.0 | Silva and Rodrigues (2001) |
| Mediterranean | <1 | Singer et al. (1996) |
| Mediterranean | <1 | Singer et al. (1996) |
| Temperate | 2.7 | Singh (1987) |
| Tropical | 1.9 | Sinun et al. (1992) |
| Mediterranean | 1.0 | Skau (1964) |
| Mediterranean | 2.0 | Skau (1964) |
| Tropical | 2.0 | Songwattana et al. (1988) |
| Temperate | <1 | Specht et al. (1957) |
| Temperate | <1 | Specht et al. (1957) |
| Temperate | 0.5 | Spittlehouse (1998) |
| Temperate | 1.0 | Spittlehouse (1998) |
| Subtropical | 5.5 | Srivastava and Ambasht (1994) |
| Temperate | 7.9 | Staelens (2006) |
| Tropical | 1.0 | Steinhardt (1979) |
| Tropical | 0.5 | Takahashi et al. (1983) |
| Tropical | 1.5 | Tanaka et al. (2005) |
| Tropical | 0.9 | Tangtham (1973) |
| Tropical | 0.3 | Tani et al. (2003) |
| Temperate | 1.2 | Taniguchi et al. (1996) |
| Temperate | 0.5 | Taniguchi et al. (1996) |
| Mediterranean | 3.3 | Thurow et al. (1987) |
| Subtropical | 0.2 | Tian et al. (1994) |
| Boreal | 0.0 | Toba and Ohta (2005) |
| Temperate | 2.7 | Toba and Ohta (2005) |
| Boreal | 0.0 | Toba and Ohta (2005) |
| Temperate | 2.5 | Toba and Ohta (2005) |
| Temperate | 3.0 | Toba and Ohta (2005) |
| Temperate | 7.0 | Tsakov and Alexandrov (2005) |
| Tropical | 19.0 | Twilley and Chen (1998) |
| Tropical | 1.4 | Ubarana (1996) |
| Temperate | 2.0 | Uyttendaele and Iroume (2002) |
| Temperate | 22.0 | Uyttendaele and Iroume (2002) |
| Mediterranean | 1.7 | Valente et al. (1997) |
| Mediterranean | 0.3 | Valente et al. (1997) |
| Boreal | <1 | Van Cleve et al. (1983) |
| Boreal | <1 | Van Cleve et al. (1983) |
| Subtropical | 0.2 | Van Stan et al. (2017) |
| Temperate | 5.3 | Van Stan (2012) |
| Temperate | 0.9 | Van Stan (2012) |
| Tropical | 0.1 | Veneklaas and Van Ek (1990) |
| Tropical | 0.8 | Vernimmen et al. (2007) |
| Tropical | 2.0 | Vernimmen et al. (2007) |
| Tropical | 1.3 | Vernimmen et al. (2007) |
| Temperate | 0.1 | Verry and Timmons (1977) |
| Temperate | 3.2 | Verry and Timmons (1977) |
| Temperate | 0.5 | Viville et al. (1993) |
| Temperate | 9.6 | Voigt (1960) |
| Temperate | 1.2 | Voigt (1960) |
| Temperate | 5.9 | Voigt (1960) |
| Tropical | 2.8 | Weaver (1972) |
| Tropical | 9.6 | Weaver (1972) |
| Tropical | 5.1 | Weaver (1972) |
| Temperate | 4.6 | Wei and Zhou (1991) |
| Temperate | 3.2 | Wheater et al. (1987) |
| Temperate | <1 | Wilm and Niederhof (1941) |
| Tropical | 0.2 | Witthawatchutikui and Tangtham (1987) |
| Subtropical | 1.9 | Yan et al. (2003) |
| Temperate | 1.8 | Xiao et al. (2007) |
| Mediterranean | 1.0 | Young et al. (1984) |
| Temperate | 2.4 | Zabret and Sraj (2015) |
| Temperate | 0.1 | Zabret and Sraj (2015) |
| Tropical | 3.0 | Zeng (1994) |
| Tropical | 0.8 | Zhang et al. (2015) |
| Tropical | 1.0 | Zhang et al. (2015) |
| Tropical | 0.8 | Zhang et al. (2015) |
| Temperate | 0.9 | Zhongjie et al. (2010) |
| Boreal | 3.3 | Zhou (2003) |

**Table S3**. Ratios of electrical conductivity between stemflow and rainfall for different forests with differing percentages of rainfall drained as stemflow (values used to generate Figure 2).

|  | Stemflow:Rainfall | |  |
| --- | --- | --- | --- |
| Species | Hydrology | EC | Study |
| *Abies alba* | 9 | 1.14 | Certini et al. (1998) |
| *Abies amabilis* | 0.4 | 8.52 | Edmonds et al. (1991) |
| *Abies balsamea* | 0.1 | 5.87 | Schmull et al. (2002) |
| *Chamaecyparis obtusa* | 12 | 1.07 | Koichiro et al. (2001) |
| *Citrus sinensis* | 4.7 | 0.60 | Li et al. (1997b) |
| *Fagus sylvatica, 03CAL1* | 1.1 | 1.96 | Mosello et al. (2002) |
| *Fagus sylvatica, 06EM12* | 6.4 | 1.46 | Mosello et al. (2002) |
| *Fagus sylvatica, 10ABR1* | 4.1 | 1.09 | Mosello et al. (2002) |
| *Fagus sylvatica, 12PIE1* | 10.3 | 1.08 | Mosello et al. (2002) |
| *Fagus sylvatica, 20VENI* | 2 | 0.89 | Mosello et al. (2002) |
| *Floodplain* | 1.1 | 3.50 | Tobón et al. (2004) |
| *High terrace* | 0.9 | 2.70 | Tobón et al. (2004) |
| Laterite terra firme | 7 | 0.88 | Jordan (1978) |
| *Low terrace* | 1.5 | 2.40 | Tobón et al. (2004) |
| Mature tropical forest | 1.5 | 3.20 | Burbano-Garcés et al. (2014) |
| *Nothofagus betuloides* | 1.2 | 2.11 | Oyarzún et al. (2004) |
| *Picea abies, 08FR12* | 0.2 | 10.92 | Mosello et al. (2002) |
| *Picea abies, 17TRE1* | 0 | 13.17 | Mosello et al. (2002) |
| *Picea jezoensis* | 1.1 | 3.10 | Sakai et al. (2004) |
| *Picea rubens* | 0.2 | 4.83 | Schmull et al. (2002) |
| *Pinus densiflora* | 3 | 1.19 | Chiwa et al. (2003) |
| *Pinus massoniana* | 0.2 | 7.02 | Zhang et al. (1996) |
| *Pinus pseudostrobus* | 0.6 | 4.83 | Silva and Rodriguez (2001) |
| *Pinus radiata* | 22 | 1.21 | Uyttendaele and Iroumé (2002) |
| *Pseudotsuga menziesii* | 0.3 | 6.10 | Edmonds et al. (1991) |
| *Quercus castanea* | 0.5 | 7.61 | Silva and Rodriguez (2001) |
| *Quercus ilex* (LC) | 2.7 | 1.41 | Rodrigo et al. (2003) |
| *Sedimentary plain* | 0.9 | 2.70 | Tobón et al. (2004) |
| *Thuja plicata* | 0.3 | 4.57 | Edmonds et al. (1991) |
| *Tsuga diversifolia* | 0.4 | 3.41 | Sakai et al. (2004) |
| *Tsuga heterophylla* | 0.5 | 4.92 | Edmonds et al. (1991) |

**References**

Aboal, J.R., Jimenez, M.S., Morales, D. and Hernandez, J.M. (2002) Net below canopy fluxes in Canarian laurel forest canopies. *Journal of Hydrology*, **264**, 201-212.

Abrahamsen, G., Horntvedt, R. and Tveite, B. (1977) Impacts of acid precipitation on coniferous forest ecosystems. *Water Air and Soil Pollution*, **8**, 57-73.

Aherne, J., Cummins, T. and Farrell, E.P. (1999) Modelling soil water fluxes in a Norway spruce {*Picea abies* (L.) Karst.} stand at Ballyhooly, Co Cork. *Irish Forestry*, **56**, 22-27.

Akpo, L.-E., Goudiaby, V.A., Grouzis, M. and Le Houerou, H.-N. (2005) Tree shade effects on soils and environmental factors in a Savanna of Senegal. *West African Journal of Applied Ecology*, **7**, 41-52.

Alvera, B. (1976) Contribucion al estudio de la intercepcion de las precipitaciones atmosfericas en el pinar de san juan de la pena. *Publicaciones del Centro Pirenaico de Biologia Experimental*, **7**, 95-100.

Anderson, A.R. and Pyatt, D.G. (1986) Interception of precipitation by pole-stage Sitka spruce and lodgepole pine and mature Sitka spruce at Kielder Forest, Northumberland. *Forestry*, **59**, 29-38.

Andersson, T. (1991) Influence of stemflow and throughfall from common oak (*Quercus robur*) on soil chemistry and vegetation patterns. *Canadian Journal of Forest Research*, **21**, 917-924.

Asdak, C., Jarvis, P.G., van Gardingen, P. and Fraser, A. (1998) Rainfall interception loss in unlogged and logged forest areas of Central Kalimantan, Indonesia. *Journal of Hydrology*, **206**, 237-244.

Aussenac, G. (1968) Interception des precipitations par le couvert forestier. *Annals of Forest Science*, **25**, 135-156.

Awasthi, O.P., Sharma, E. and Palni, M.S. (1995) Stemflow: A source of nutrients in some naturally growing epiphytic orchids of the Sikkim Himalaya. *Annals of Botany*, **75**, 5-11.

Badri, W. and Gauquelin, T. (2003) The hydrological cycle and changes of soil water storage in a Thuriferous Juniper (*Juniperus thurifera* L.) stand in the Moroccan High Atlas Mountains. International Conference on Ecohydrology of High Mountain Areas, Part C: Atmospheric, Hydrologic, and Ecological Interactions, 315-321.

Bahmani, S.M.H.G., Attarod, P., Bayramzadeh, V., Ahmadi, M.T., and Radmehr, A. (2012) Throughfall, stemflow, and rainfall interception in a natural pure forest of chestnut-leaved oak (*Quercus castaneifolia* C.A. Mey.) in the Caspian Forest of Iran. *Annals of Forest Research*, **55**, 197-206.

Baker, J., Hocking, D. and Nyborg, M. (1977) Acidity of open and intercepted precipitation in forests and effects on forest soils in Alberta, Canada. *Water Air and Soil Pollution*, **7**, 449-460.

Belli, M., Tikhomirov, A., Kliashtorin, A., Shcheglov, A., Rafferty, B., Shaw, G., Wirth, E., Kammerer, L., Ruehm, W., Steiner, M., Delvaux, B., Maes, E., Kruyts, N., Bunzl, K., Dvornik, A.M. and Kuchma, N. (1996) In A. Karaoglou, G. Desmet, G.N. Kelly and H.G. Menzel (Eds.) *The radiological consequences of the Chernobyl accident* (pp 69-80). Brussels, Belgium: European Commission.

Bergkvist, B. and Folkeson, L. (1995) Soil acidification and element fluxes of a *Fagus sylvatica* forest as influenced by simulated nitrogen deposition. *Water Air and Soil Pollution*, **65**, 111-133.

Bittner, S., Talkner, U., Kraemer, I., Beese, F., Hoelscher, D. and Priesack, E. (2010) Modeling stand water budgets of mixed temperate broad-leaved forest stands by considering variations in species specific drought response. *Agricultural and Forest Meteorology*, **150**, 1347-1357.

Brauman, K.A., Freyberg, D.L. and Daily, G.C. (2010) Forest structure influences on rainfall partitioning and cloud interception: A comparison of native forest sites in Kona Hawai’i. *Agricultural and Forest Meteorology*, **150**, 265-275.

Brown, M.B., de la Roca, I., Vallejo, A., Ford, G., Casey, J., Aguilar, B. and Haacker, R. (1996) A Valuation Analysis of the Role of Cloud Forests in Watershed Protection. Sierra de las Minas Biosphere Reserve, Guatemala and Cusuco National Park, Honduras. RARE Center for Tropical Conservation, Philadelphia.

Bryant, M.L., Bhat, S. and Jacobs, J.M. (2005) Measurements and modeling of throughfall variability for five forest communities in the southeastern US. *Journal of Hydrology*, **312**, 95-108.

Burbano-Garcés, M.L., Figueroa-Casas, A. and Peña, M. (2014) Bulk precipitation, throughfall and stemflow deposition of N-NH4+, N-NH3 and N-NO3- in an Andean forest. *Journal of Tropical Forest Science*, **26**, 446-457.

Calabuig, E.L., Gago Gamallo, M.L. and Gomez Gutierrezm J.M. (1978) Influencia de la encina (*Quercus rotundifolia* Lam.) en la ditribucion del agua de lluvia. *Anuario del Centro de Edafologia y Biologia Aplicada de Salamanca*, **4**, 143-159.

Cape, J.N., Brown, A.H.F., Robertson, S.M.C., Howson, G. and Paterson, I.S. (1991) Interspecies comparisons of throughfall and stemflow at three sites in northern Britain. *Forest Ecology and Management*, **46**, 165-177.

Carlisle, A., Brown, A.H.F. and White, E.J. (1967) The nutrient content of tree stem flow and ground flora litter and leachates in a sessile oak (*Quercus petraea*) woodland. *Journal of Ecology*, **55**, 615-627.

Cavalier, J., Jaramillo, M., Solis, D. and de Leon, D. (1997) Water balance and nutrient inputs in bulk precipitation in tropical montane cloud forest in Panama. *Journal of Hydrology*, **193**, 83-96.

Cecchi, G.A., Kröpfl, A.I., Villasuso, M. and Distel, R.A. (2006) Stemflow and soil water redistribution in intact and disturbed plants of *Larrea divaricate* in Southern Argentina. *Arid Land Research and Management*, **20**, 209-217.

Certini, G., Ugolini, F.C., Corti, G. and Agnelli, A. (1998) Early stages of podsolization under Corsican pine (*Pinus nigra* Arn. *ssp. laricio*). *Geoderma*, **83**, 103-125.

Chang, S.-C. and Matzner, E. (2000) The effect of beech stemflow on spatial patterns of soil solution chemistry and seepage fluxes in a mixed beech/oak stand. *Hydrological Processes*, **14**, 135-144.

Chappell, N.A., Bidin, K. and Tych, W. (2001) Modelling rainfall and canopy controls on net-precipitation beneath selectively-logged tropical forest. *Plant Ecology*, **153**, 215-229.

Charoensuk, S., Jirasuktaveekul, W. and Onarsa, S. (1989) Rainfall intercepted by teak plantation. In A. Nalanpoon, P. Thitirojanawat and P. Tippharaot (Eds.), *Abstract of Research Works in Watershed Management in Thailand* (#39). Bangkok, Thailand: Forest Environment Research and Development Division, Forest Research Office, Royal Forest Department.

Chiwa, M., Kim, D.H. and Sakugawa, H. (2003) Rainfall, stemflow, and throughfall chemistry at urban- and mountain-facing sites at Mt. Gokurakuji, Hiroshima, Western Japan. *Water Air and Soil Pollution*, **146**, 93-109.

Chunkao, K., Tangtham, N. and Ungkulpakdikul, S. (1971) Measurements of rainfall in early wet season under hill-and-dry evergreen, natural teak, and dry depteterpcarp forests of Thailand. *Kog-Ma Watershed Research Bulletin*, **10**, 1-31.

Chunpaga, U. and Wachirajutipong, T. (1977) Interception, throughfall and stemflow of mixed deciduous with Teak forest. In A. Nalanpoon, P. Thitirojanawat and P. Tippharaot (Eds.), *Abstract of Research Works in Watershed Management in Thailand* (#27). Bangkok, Thailand: Forest Environment Research and Development Division, Forest Research Office, Royal Forest Department.

Chuyong, G.B., Newberry, D.M. and Songwe, N.C. (2004) Rainfall input, throughfall and stemflow of nutrients in a central African rain forest dominated by ectomycorrhizal trees. *Biogeochemistry*, **67**, 73-91.

Courchesne, F. and Hendershot, W.H. (1988) Supply of sulphate and water at the soil surface under four tree species. *Naturaliste Canadien*, **115**, 57-63

Cowling, R.M. and Mills, A.J. (2011) A preliminary assessment of rain throughfall beneath *Portulacaria afra* canopy in subtropical thicket and its implications for soil carbon stocks. *South African Journal of Botany*, **77**, 236-240.

Crockford, R.H. and Richardson, D.P. (1990) Partitioning of rainfall in a eucalypt forest and pine plantation in southeastern Australia: IV the relationship of interception and canopy storage capacity, the interception of these forests, and the effect on interception of thinning the pine plantation. *Hydrological Processes*, **4**, 169-188.

David, T.S., Gash, J.H.C., Valente, F., Pereira, J.S., Ferreira, M.I. and David, J.S. (2006) Raingfall interception by an isolated evergreen oak tree in a Mediterranean savannah. *Hydrological Processes* , **20**, 2713-2726.

Delfs, J. (1967) Interception and stemflow in stands of Norway spruce and beech in West Germany. In *International Symposium of Forest Hydrology* (pp. 179-185). New York, USA: Pergamon Press.

Dewalle, D.R. and Paulsell, L.K. (1979) Canopy interception, stemflow and streamflow on a small drainage in the Missouri Ozarks. *Research Bulletin of the University of Missouri*, **951**.

Dezzeo, N. and Chacón, N. (2006) Nutrient fluxes in incident rainfall, throughfall, and stemflow in adjacent primary and secondary forests of the Gran Sabana, southern Venezuela. *Forest Ecology and Management*, **234**, 218-226.

Didon-Lescot, J.-F. (1996) Forêt et développement durable au Mont Lozère. Dissertation thesis: Univerisity of Orlèans.

Dolman, A.J. (1987) Summer and winter rainfall interception in an oak forest. Predictions with an analytical and a numerical simulation model. *Journal of Hydrology*, **90**, 1-9.

Domingo, F., Puigdefabregas, J., Moro, M.J. and Bellot, J. (1994) Role of vegetation cover in the biogeochemical balances of a small afforested catchment in southeastern Spain. *Journal of Hydrology*, **159**, 275-289.

Dolegowska, S. and Migaszewski, Z.M. (2013) Anomalous concentrations of rare earth elements in the moss-soil system from south-central Poland. *Environmental Pollution*, **178**, 33-40.

Dovey, S.B., du Toit, B. and de Clercq, W. (2011) Nutrient fluxes in rainfall, throughfall and stemflow in *Eucalyptus* stands on the Zululand coastal plain, South Africa. *Southern Forests*, **73**, 193-206.

Dunin, F., O’Loughlin, E., and Reyenga, W. (1988) Interception loss from eucalypt forest: lysimeter determination of hourly rates for long term evaluation. *Hydrological Processes*, **2**, 315-329.

Durocher, M.G. (1990) Monitoring spatial variability of forest interception. *Hydrological Processes*, **4**, 215-229.

Dykes, A.P. (1997) Rainfall interception from a lowland tropical rainforest in Brunei. *Journal of Hydrology*, **200**, 260-279.

Edmonds, R.L., Thomas, T.B. and Rhodes, J.J. (1991) Canopy and soil modification of precipitation chemistry in a temperate rain forest. *Soil Science Society of America Journal*, **55**, 1685-1693.

Edwards, P.J. (1982) Studies of mineral cycling in a montane rain forest in New Guinea. *Journal of Ecology*, **70**, 807-827.

Ellsbury, M.M., Jackson, J.J., Woodson, W.D., Beck, D.L. and Stange, K.A. (1996) Efficacy, application distribution, and concentration by stemflow of *Steinernema carpocapsae* (Rhabditida: Steinernematidae) suspensions applied with a lateral-move irrigation system for corn rootworm (Coleoptera: Chrysomelidae) control on maize. *Biological and Microbial Control*, **89**, 74-81.

Eren, M. and Hatipoglu-Bagci, Z. (2010) Karst surface features of the hard laminated crust (Caliche Hardpan) in the Mersin Area, Southern Turkey. *Acta Carsologica*, **39**, 93-102.

Fan, H.B. and Hong, W. (2001) Estimation of dry deposition and canopy exchange in Chinese fir plantations. *Forest Ecology and Management*, **147**, 99-107.

Fathizadeh, O., Attarod, P., Pypker, T.G., Darvishsefat, A.A. and Zahedi Amiri, G. (2013) Seasonal variability of rainfall interception and canopy storage capacity measured under individual oak (*Quercus brantii*) trees in western Iran. *Journal of Agricultural Science and Technology*, **15**, 175-188.

Ferreira, A.J.D. (1992) Variáveis hidro-meteorológicas em povoamentos de *Pinus pinaster* e *Eucalyptus globulus*. *Um caso de estudo, VI Colóquio Ibérico de Geografia-Actas* (pp. 935–941). A Península Ibérica-um espaço em mutaçaõ.

Fleischbein, K., Wilcke, W., Valarezo, C., Zech, W. and Knoblich, K. (2006) Water budgets of three small catchments under montane forest in Ecuador: experimental and modelling approach. *Hydrological Processes*, **20**, 2491-2507.

Foster, N.W. (1974) Annual microelement transfer from *Pinus banksiana* Lamb. Forest to soil. *Canadian Journal of Forest Research*, **4**, 470-476.

Frangi, J.L., Barrera, M.D., Puigdefábregas, J., Yapura, P.F., Arambarri, A.M. and Richter, L.L. (2005) Ecologia de los bosques de Tierra Fuego. In J.F. Goya, J.L. Frangi and M.F. Arturi (Eds.), *Ecologia y Manejo de los Bosques de Argentina* (pp. 1-88). Buenos Aires, Argentina: Editorial de la Universidad Nacional de La Plata.

Freedman, B. and Prager, U. (1986) Ambient bulk deposition, throughfall, and stemflow in a variety of forest stands in Nova Scotia. *Canadian Journal of Forest Research*, **16**, 854-860.

Gascon, A.F. (1994) Nutrient returns and some hydrologic characteristics of *Coffea robusta* L. (Rub.) + *Gmelina arborea* Roxb. and *Theobroma cacao* L. + *Gmelina arborea* Roxb. agroforestry systems in Mt. Makiling (Philippines). Laguna, Philippines: Philippines University.

Gash, J.H.C. and Morton, A.J. (1978) An application of the Rutter Model to the estimation of the interception loss from Thetford Forest. *Journal of Hydrology*, **38**, 49-58.

Gerold, G., Schawe, M. and Bach, K. (2008) Hydrometeorologic, pedologic and vegetation patterns along an elevational transect in the montane forest of the Bolivian Yungas. *Die Erde*, **139**, 141-168.

Ghimire, C.P., Bruijnzeel, L.A., Lubczynski, M.W. and Bonell, M. (2012) Rainfall interception by natural and planted forests in the Middle Mountains of Central Nepal. *Journal of Hydrology*, **475**, 270-280.

Ghimire, C.P., Bruijnzeel, L.A., Lubczynski, M.W., Ravelona, M., Zwartendijk, B.W. and van Meerveld, H.J. (2017) Measurement and modeling of rainfall interception by two differently aged secondary forests in upland eastern Madagascar. *Journal of Hydrology*, **545**, 212-225.

Ghorbani, S. and Rahmani, R. (2009) Estimating of interception loss, stemflow and throughfall in a natural stand of oriental beech (Shastkalateh Forest). *Iranian Journal of Forest and Poplar Research*, **16**, 638-638.

Giacomin, A. and Trucchi, P. (1992) Rainfall interception in a beech coppice (Acquerino, Italy). *Journal of Hydrology*, **137**, 141-147.

Gill, D. (1975) Influence of white spruce trees on permafrost-table microtopography, Mackenzie River Delta. *Canadian Journal of Earth Sciences*, **12**, 263-272.

Godoy, R., Oyarzun, C. and Bahamondes, J. (1999) Flujos hidroquímicos en bosque de *Nothofagus pumilio* en el Parque Nacional Puyehue, sur de Chile. *Revista de Historia Natural*, **72**, 579-594.

Gómez, J.A., Vanderlinden, K., Giráldez, J.V. and Fereres, E. (2002) Rainfall concentration under olive trees. *Agricultural Water Management*, **55**, 53-70.

Gomez-Peralta, D., Oberbauer, S.F., McClain, M.E. and Philippi, T.E. (2008) Rainfall and cloud-water interception in tropical montane forests in the eastern Andes of Central Peru. *Forest Ecology and Management*, **255**, 1315-1325.

Gönczöl, J. and Révay, A. (2006) Species diversity of rainborne hyphomycete conidia from living trees. *Fungal Diversity*, **22**, 37-54.

González-Cascón, M>R., Arias, M.L., Javita, M.S. and Gallego, M.T.M. (1994) Balance de entradas/salidas de cations en una pequeña Cuenca forestall de *Pinus sylvestris* en la Sierra de Guadarrama. *Ecología*, **8**, 157-166.

Gupta, A. and Usharani, L. (2009) Rainfall partitioning in a tropical forest of Manipur, North East India. *Tropical Ecology*, **50**, 355-358.

Hakimi, L., Sadeghi, S.M.M. and Khosropur, I. (2017) Rainfall partitioning into throughfall, stemflow, and interception of Pomegranate and its importance in ecohydrology. *Iran Water Resources Research*, in press.

Hall, R.L., Calder, I.R., Nimal Gunawardena, E.R. and Rosier, P.T.W. (1996) Dependence of rainfall interception on drop size: 3. Implementation and comparative performance of the stochastic model using data from a tropical site in Sri Lanka. *Journal of Hydrology*, **185**, 389-407.

Hauck, M.and Spribille, T. (2005) The significance of precipitation and substrate chemistry for epiphytic lichen diversity in spruce-fir forests of the Salish Mountains, Northwestern Montana. *Flora*, **200**, 547-562.

Helvey, J.D. (1967) Interception by eastern white pine. *Water Resources Research*, **3**, 723-729.

Herwitz, S.R. (1986) Infiltration-excess caused by stemflow in a cyclone-prone tropical rainforest. *Earth Surface Processes and Landforms*, **11**, 401-412.

Hildebrandt, A. and Eltahir, E.A.B. (2006) Forest on the edge: Seasonal cloud forest in Oman creates its own ecological niche. *Geophysical Research Letters*, **33**, L11401.

Hölscher, D., Köhler, L., Leuschner, C. and Kappelle, M. (2003) Nutrient fluxes in stemflow and throughfall in three successional stages of an upper montane rainforest in Costa Rica. *Journal of Tropical Ecology*, **19**, 557-565.

Hoppe (1896) Regenmessung unter Baumkrone (Rain measurement under tree tops). *Mitt. Aus dem forstlichen Versuchswegen Oesterreichs (Conclusions from the Austrian* *Forestry Experimentation)*, **21**, 1-75.

Hosseini, G.B.S.M., Attarod, P., Hossein, B., Vilma, B., Hamid, S.M. and Mostafa, J.B. (2011) Stemflow generations in a pure oak forest stand within the growing season. *Research Journal of Forest Science and Engineering*, **1**, 25-53.

Hutjes, R.W.A., Wierda, A. and Veen, A.W.L. (1990) Rainfall interception in the Tai Forest, Ivory Coast: Application of two simulation models to a humid tropical system. *Journal of Hydrology*, **114**, 259-275.

Ibrahim, M., Rapp, M. and Lossaint, P. (1982) Economie de l’eau d’un écosystème à *Pinus pinea* L. du littoral méditerranéen. *Annals of Forest Science*, **39**, 289-306.

Iida, S.-I., Tanaka, T. and Sugita, M. (2005) Change of interception process due to the succession from Japanese red pine to evergreen oak. *Journal of Hydrology*, **315**, 154-166.

Iovino, F., Cinnirella, S., Veltri, A. and Callegari, G. (1998) Processus hydriques dans des écosystèmes forestiers. *Écologie*, **29**, 1-2.

Jackson, J. (1971) Problems of throughfall and interception assessment under tropical forest. *Journal of Hydrology*, **12**, 234-254.

James, E. and Pradeepkumar, P. (1987) Studies on the hydrological processes in the forest drainage basins of the Western Ghats of India. *Forest Hydrology and Watershed Management*, **167**, 223-229.

Jetten, V. (1996) Interception of tropical rainforest: Performance of a canopy water balance model. *Hydrological Processes*. **10**, 671-685.

Jirasuktaveekul, W., Charoensuk, S. and Witthawatchutikul, P. (1987) Rainfall intercepted by *Melia azedarach* Linn. plantation. In A. Nalanpoon, P. Thitirojanawat and P. Tippharaot (Eds.), *Abstract of Research Works in Watershed Management in Thailand* (#29). Bangkok, Thailand: Forest Environment Research and Development Division, Forest Research Office, Royal Forest Department.

Johnson, R. (1990) The interception, throughfall, and stemflow in a forest in highland Scotland and the comparison with other uplan forests in the U.K. *Journal of Hydrology*, **118**, 281-287.

Jordan, C.F. (1978) Stem flow and nutrient transfer in a tropical rain forest. *Oikos*, **31**, 257-263.

Kabral, N., and Frey, J. (2009) Heavy metal accumulation in different parts of coniferous forest. In L. Ukunmaanaho, T.M. Nieminen, and M. Starr (Eds.) *Proceedings of the 6th International Symposium on Ecosystem Behaviour BIOGEOMON*. Working Papers of the Finnish Forest Research Institute, **128**, 379.

Kelliher, F.,Whitehead D. and Pollock, D. (1992) Rainfall interception by trees and slash in a young Pinus radiata D. Don stand. *Journal of Hydrology*, **131**, 187-204.

Kellman, M. and Roulet, N. (1990) Stemflow and throughfall in a tropical dry forest. *Earth Surface Processes and Landforms*, **15**, 55-61.

Khan, M.A. (1991) Water balance and hydrochemistry of precipitation components in forested ecosystems in the arid zone of Rajasthan, India. *Hydrological Sciences Journal*, **44**, 149-161.

Kirkpatrick, J.B. (1997) Vascular plant-eucalypt interactions. In J. Williams and J. Woinarski (Eds.), Eucalypt Ecology: Individuals to Ecosystems (pp. 227-245). Cambridge, UK: Cambridge University Press.

Klučiarová, D., Márton, P., Pitchler, V., Márton, and E., Túnyi, I. (2008) Pollution detection by magnetic susceptibility measurements aided by the stemflow effect. *Water Air Soil Pollut*, **189**, 213- 223.

Krämer, I. and Höscher, D. (2009) Rainfall partitioning along a tree diversity gradient in a deciduous old-growth forest in central Germany. *Ecohydrology*, **2**, 102-114.

Křeček, J., Palán, L., and Stuchlík (2017) Acid atmosphere deposition in a forested mountain catchment. *iForest Biogeosciences and Forestry*, **10**, 680-686.

Krishna, R.B., Kurothe, R.S., Pande, V.C. and Kumar, G. (2012) Throughfall and stemflow measurement in bamboo (*Dendrocalmus strictus*) plantation. *Indian Journal of Soil Conservation*, **40**, 60-64.

Koichiro, K., Tanaka, Y., Tanaka, N. and Karakama, I. (2001) Generation of stemflow volume and chemistry in a mature Japanese cypress forest. *Hydrological Processes*, **15**, 1867-1978.

Laclau, J., Ranger. J., Bouillet, J., Nzilla, J. and Deleporte, P. (2003) Nutrient cycling in a clonal stand of eucalyptus and an adjacent savanna ecosystem in Congo. *Forest Ecology and Management,* **176**, 105-119.

Lawson, E. (1967) Throughfall and stemflow in a pine-hardwood stand in the Ouachita mountains of Arkansas. *Water Resources Management*, **3**, 371-375.

Lazerjan, M.S. (2012) Hydrochemistry of rainfall and stemflow of *Juglans regia* Linn. And *Cupressus sempervirens* L. var. *Fastigiata* in the North of Iran. *Ecopersia*, **1**, 85-98.

Lee, D., Kim, G., Joo, K. and Kim, Y. (1997) Throughfall, stemflow and rainfall interception loss in Pinus koraiensis Sieb. et Zucc., Larix leptolepis (Sieb. et Zucc.) Gordon and Quercus species stand at Kwangju-gun, Kyunggi-Do. *Korean For Soc*, **86**, 200-207.

Lei, R., Zhang, Y. and Dang, K. (1994) A study on hydrological effects of forest in the Qinling Mountains Forest Region. In X. Zhou (Ed.) *Studies on Forest Ecosystems* (pp. 223-234). Harbin, China: Northeast Forestry University Press.

Li, X.-Y., Liu, L.-Y., Gao, S.-Y., Ma, Y.-J. and Yang, Z.-P. (2008) Stemflow in three shrubs and its effect on soil water enhancement in semiarid loess region of China. *Agricultural and Forest Meteorology*, **148**, 1501-1507.

Li, Y., Alva, A., Calvert D. and Zhang M (1997a) Stemflow, throughfall, and canopy interception of rainfall by citrus tree canopies. *HortScience*, **32**, 1059-1060.

Li, Y., Alva, A., Calvert D. and Zhang M (1997b) Chemical composition of throughfall and stemflow from citrus canopies. *Journal of Plant Nutrition*, **20**, 1351-1360.

Lilienfein, J. and Wilcke, W. (2004) Water and element input into native, agri- and silvicultural ecosystems of the Brazilian savanna. *Biochemistry*, **67**, 183-212.

Liu, C.P. and Sheu, B.H. (1999) Distribution and chemical characteristics of nutrients in throughfall and stemflow of three different stands. *Quarterly Journal of Forest Research*, **21**, 51-59.

Liu, S., Sun, P., Wang, J. and Chen L. (2001) Hydrological functions of forest vegetation in upper region of the Yangtze River. *Journal of Natural Resources*, **16,** 451-456.

Liu, W., Fox, J.E.D. and Xu, Z. (2002) Nutrient flux in bulk precipitation throughfall and stemflow in montane subtropical moist forest on Ailao Mountains in Yunnan, S.W. China. *Journal of Tropical Ecology*, **18**, 527-548.

Liu, W., Fox, J.E.D. and Xu, Z. (2003) Nutrient budget of a montane evergreen broad-leaved forest at Ailao Mountain National Nature Reserve, Yunnan, Southwest China. *Hydrological Processes*, **17**, 1119-1134.

Liu, W.J., Liu, W.Y., Li, J.T., Wu, Z.W. and Li, H.M. (2008) Isotope variations of throughfall, stemflow and soil water in a tropical rain forest and rubber plantation in Xishuangbanna, SW China. *Hydrology Research*, **39**, 437-449.

Llorens, P. and Domingo, F. (2007) Rainfall partitioning by vegetation under Mediterranean conditions. A review of studies in Europe. *Journal of Hydrology*, **335**, 37-54.

Llorens, P., Poch, R., Latron, J. and Gallart, F. (1997) Rainfall interception by a *Pinus sylvestris* forest patch overgrown in a Mediterranean mountainous abandoned area I. Monitoring design and results down to the event scale. *Journal of Hydrology*, **199**, 331-345.

Lloyd, C.R. and Marques, A.D.O. (1988) Spatial variability of throughfall and stemflow measurements in Amazonian rainforest. *Agricultural and Forest Meteorology*, **42**, 63-73.

Lloyd, C.R., Shuttleworth, W.J. and Marques, A.D.O. (1988) The measurement and modelling of rainfall interception by Amazonian rain forest. *Agricultural and Forest Meteorology*, **43**, 277-294.

Lockwood, J.G. (1985) World Climate Systems. Edward Arnold, London, 292 pp.

Loshali, D.C. and Singh, R.P. (1992) Partitioning of rainfall by three Central Himalayan forests. *Forest Ecology and Management*, **53**, 99-105.

Lu, J., Zhang, S.X., Fang, J.P. and Zheng, W.L. (2016) Nutrient characteristics of throughfall and stemflow in the natural forest of *Pinus densata* in the Tibetan plateau. *Phyton - International Journal of Experimental Botany*, **85**, 142-148.

Luangjame, J., Boontawee, B. and Kliangpibool, N. (2001) Determination of deposition and leaves in teak plantations in Thailand. *Water Air and Soil Pollution*, **130**, 935-940.

Lugo, A.E., Gamble, J.F. and Ewel, K.C. (1976) Organic matter budget in a mixed-hardwood forest in north central Florida. In D.C. Adriano and I.L. Brisbin (Eds.) *Environmental Chemistry and Cycling Processes* (pp. 790-800). Augusta, Georgia, USA: US Department of Energy (CONF-760429).

Lukina, N.V. (2008) Plant-induced variability in soil acidity and nutrient stats of boreal forest ecosystems. In J. Derome, A.-J. Lindroos and T. Kilponen (Eds.) *Scientifc Seminar on Forest Condition Monitoring and Ecosystem Functioning in Northern Europe under the Forest Focus and ICP Forests programmes*. Working Papers of the Finnish Forest Research Institute, **74**, 48-53.

Luschev (1940) Osadki pod pologom lesa (Precipitation under forest canopy). In: Sbornik Vodookhrannaya rol' lesa. Izdatel'stvo, VNIILKh.

Mahendrappa, M.K. (1974) Chemical composition of stemflow from some Eastern Canadian tree species. *Canadian Journal of Forest Research*, **4**, 1-7.

Mahendrappa, M.K. and Ogden, E. (1973) Effects of fertilization of a black spruce stand and nitrogen contents of stemflow, throughfall, and litterfall. *Canadian Journal of Forest Research*, **3**, 54-60

Mair, A. and Fares, A. (2010) Throughfall characteristics in three non-native hawaiian forest stands. *Agricultural and Forest Meteorology*, **150**, 1453–1466.

Majima, M. and Tase, N. (1982) Spatial variation of rainfall in a red pine forest. *Bulletin of Environmental Research Center University of Tsukuba*, **6**, 75-82.

Malone, M.S. (2015) Hydrological and biogeochemical fluxes of throughfall and stemflow in temperate swamps. Master of Science Thesis, University of Toronto.

Manfroi, O., Koichiro, K., Suzuki, M., Tanaka, N., Kume, T., Nakagawa, M., Kumaigai, T., and Nakashizuka, T. (2006) Comparison of conventionally observed interception evaporation in a 100-m^2^ subplot with that estimated in a 4-ha area of the same Bornean lowland tropical forest. *Journal of  Hydrology*, **329**, 329–349.

Manokaran, N. (1979) Stemflow, throughfall and rainfall interception in a lowland tropical rain forest in peninsular Malaysia. *Malaysian Forester*, **42**, 174-201.

Marin, C.T., Bouten, I., and Dekker, S. (2000) Forest floor water dynamics and root water uptake in four forest systems in northwest Amazonia. *Journal of Hydrology*, **237**, 169–183.

Mateos, B. and Schnabel, S. (1998) Medición de la intercepción de las precipitaciones por la encina (Quercus rotundifolia Lam.): Metodología y primeros resultados. In: Gómez-Ortiz, A., Salvador, F. (Eds.), Investigaciones recientes de la Geomorfología Española, Barcelona, 529–538.

Mauchamp, A. and Janeau, J.  (1993) Water funnelling by the crown of Flourensia cernua, a Chihuahuan desert shrub. *Journal of Arid Environments*, **25**, 299-306

Melovski, L.,  Grupce, L., and Mulev, M. (1992) Nutrient Cycling Quercetum Frainetto-Cerris Macedonicum Ecosystem In Galicica National Park. In A. Teller, P. Mathy, and J.N.R. (Eds.) *Responses of Forest Ecosystems to Environmental Changes* (pp. 405-416). Brussels, Belgium: Springer.

Michopoulos, P., Baloutsos, G., Nakos, G., and Economou, A. (2001) Effects of bulk rainfall pH and growth period on cation enrichment in rainfall beneath the canopy of a beech (Fagus moesiaca) forest stand. *The Science of the Total Environment*, **281**, 79-85

Miole, R., Visco, R., Magcale-Macandog, D., Abucay, E., Gascon, A., and Castillo, A. (2011) Growth performance, crop productivity, and water and nutrient flows in *Gmelina arborea*Roxb.-*Zea mays* hedgerow systems in Southern Philippines. *Philippine Journal of Crop Science*, **36**, 34–44

Molchanov, A.A. (1960) The Hydrological Role of Forests. Academy of Science of the U.S.S.R. Institute of Forestry, 407 pp.

Monokaran, N. (1979) Stemflow throughfall and rainfall interception in a lowland tropical rain forest in peninsular Malaysia. *The Malaysian Forester*, **42**, 174-201

Moore, T. (1980) The nutrient status of subartic woodland soils*. Artic and Alpine Research*, **12**, 147-169

Moore, T. (2003) Dissolved organic carbon in a northern boreal landscape. *Global Biogeochemical Cycles*, **17**, 1109-1117

Moreno, G., Gallardo, J., and Menéndez, I. (1993) Water and bioelement ﬂuxes in a Castanea sativa forest In *Proceedings of the International Congress on Chestnut* (pp. 247-250). Spoleto, Italy: International Society for Horticultural Science.

Moreno, M., Gallardo, J. F., and Bussotti, F. (2001). Canopy modification of atmospheric deposition in oligotrophic *Quercus pyrenaica* forests of an unpolluted region (central-western Spain). *Forest Ecology and Management,* ***149***, 47–60

Mosello, R., Brizzio, M.C., Kotzias, D., Marchetto, A., Rembges, D., and Tartari, G. (2002) The chemistry of atmospheric deposition in Italy in the framework of the National Programme for Forest Ecosystems Control (CONECOFOR). *Journal of Limnology*, **61**, 77–92

Munishi, P. and Shear, T.H. (2005) Rainfall interception and partitioning in afromontane rainforests of the eastern arc mountains, Tanzania: Implications for water conservation. *Journal of Tropical Forest Science*, **17**, 355-365.

Murai, H. (1970) Studies on precipitation interception by forest vegetation. *Bull Gov For Exp Stat*,

**232**, 25-64.

Návar, J. (1993) The causes of stemflow variation in three semi-arid growing species of northeastern Mexico. *Journal of Hydrology*, **145**, 175-190.

Návar, J. and Bryan, R. (1990) Interception loss and rainfall redistribution by three semi-arid shrubs in northeastern Mexico. *Journal of Hydrology*, **115**, 51-63

Neal, C., Robson, A., Bhardwaj, C., Conway, T., Jeffery, H., Neal, M., Ryland, G., Smith, C., and Walls, J. (1993) Relationships between precipitation, stemﬂow and throughfall for a lowland beech plantation, Black Wood, Hampshire, southern England. Findings on interception at a forest edge and the effects of storm damage. *Journal of Hydrology*, **146**, 221–233

Neary, A. and Gizyn, W.  (1994) Throughfall and stemflow under deciduous and coniferous forest canopies in south-central Ontario. *Canadian Journal of Forest Research,* **24**, 1089-1110

Negi, G., Rikhari, H., and Garkoti, S. (1998) The hydrology of three high-altitude forests in central Himalaya, India: A reconnaissance study. *Hydrologic Processes*, **12**, 343-350

Nihlgård, B. (1970) Precipitation, Its Chemical Composition and Effect on Soil Water in a Beech and a Spruce Forest in South Sweden. *Nordic Society Oikos*, **21**, 208-217

Nizinski, J., Morand, D., and Saugier, B. (1989) Variation of stomatal resistance with leaf age in Quercus petraea: effect on the soil-water balance of an oak forest. *Annals of Forest Science*,**46**, 429-432

Noirfalise A. (1959) Sur l’interception de la pluie par le couvert dans quelques forêts belges. *Bull. Soc. Roy. For. Belgique*, **10**, 433–439

Nulsen, R., Bligh, K., Baxter, I., Imrie, D. (1986) The fate of rainfall in a mallee and heath vegetated catchment in southern Western Australia. *Australian Journal of Ecology*, **11**, 361–371

Onarsa, S. (1995) Rainfall interception of *Leuocena leucouphara* Lam. De. Wit. at Pasak Watershed Research Station, Pechabun province. In A. Nalanpoon, P. Thitirojanawat and P. Tippharaot (Eds.), *Abstract of Research Works in Watershed Management in Thailand* (#55). Bangkok, Thailand: Forest Environment Research and Development Division, Forest Research Office, Royal Forest Department.

Opakunle, J.  (1989) Throughfall, stemflow and rainfall interception in a Cacao plantation in South Western Nigeria. *Tropical Ecology*, **30**, 244-252

Orr, H.K. (1972) Throughfall and stemflow relationships in second growth ponderosa pine in the Black Hills. Rocky Mountain Forest and Range Experiment Station, Research Note Rm-210. Ft. Collins, Colorado: USDA Forest Service.

Orvington, J. (1954) A comparison of rainfall in different woodlands. *Forestry*, **27**, 41-53.

Owens, M., Lyons, R., and Alejandro, C. (2006) Rainfall partitioning within semi-arid juniper communities: effects of event size and canopy cover. *Hydrologic Processes*, **20**, 3179-3189

Oyarzún, C.E., Godoy, R., de Schrijver, A., Staelens, J. and Lust, N. (2004) Water chemistry and nutrient budgets in an undisturbed evergreen rainforest of southern Chile. *Biogeochemistry*, **71**, 107-123.

Pathak, P., Pandey, A. and Singh, J. (1985) Apportionment of rainfall in central Himalayan forests (India). *Journal of Hydrology*, **76**, 319—332.

Patric, J. (1966) Rainfall interception by mature coniferous forests of Southeast Alaska. *Journal of Soil and Water Conservation*, **21**, 229-231.

Paungchareon, (1987) Rainfall interception in *Acasia auriculaeformis* plantation. In A. Nalanpoon, P. Thitirojanawat and P. Tippharaot (Eds.), *Abstract of Research Works in Watershed Management in Thailand* (#30). Bangkok, Thailand: Forest Environment Research and Development Division, Forest Research Office, Royal Forest Department.

Petit, F. and Kamuntanda. K. (1984) Interception des pluies par différents types de couverts forestiers. *Bull de la Soc Géog de Liège*, **20**, 99-127.

Piirainen, S., Finér, L., and Starr, M. (1998) Canopy and soil retention of nitrogen deposition in a mixed boreal forest in eastern Finland. *Water and Air Soil Pollution*, **105**, 165–174.

Pipkov, N., Zhelev, P. and Draganova, I. (2000) Proceedings of the scientific conference *Юбилеен сборник научни доклади: 75 години висше лесотехническо образование в Б"лгария. Секция Екология и опазване на околната среда* (Jubilee collection of scientific papers: 75 years of higher forestry education in Bulgaria) (pp. 331-340). Sofia, Bulgaria: Section of Ecology and Environmental Protection.

Plamondon, A.., Prévost, M. and Naud, R. (1984) Interception de la pluie dans la sapinière à bouleau blanc, Forêt Montmorency. *Canadian Journal for Forest Research,* **14**, 722.

Portela, E., and Pires, A.L. (1995) Nutrient deposition and leaching by rainwater in low and intensitively managed chestnut groves. In *Proceedings of the Conference on Erosion and Land Degradation in the Mediterranean* (pp. 307-317). Aveiro, Portugal: University of Aveiro.

Prebble, R. and Stirk, G. (1980)  Throughfall and stemflow on silverleaf ironbark (*Eucalyptus melanophloia*) trees. *Austral Ecology*, **5**, 419-427.

Pressland, A. (1973) Rainfall partitioning by an arid woodland (Acacia aneura F. Muell.) in south-western Queensland. *Australian Journal of Botany*, **21**, 235-245.

Price, A. and Carlyle-Moses, D. (2003) Measurement and modeling of growing-season canopy water fluxes in a mature mixed deciduous forest stand, south Ontario, Canada. *Agricultural Forest Meteorology*, **119**, 69–85.

Price, A., Dunham K., Carleton T., and Band L. (1997) Variability of water fluxes through the black spruce (Picea mariana) canopy and feather moss (Pleurozium schreberi) carpet in the boreal forest of northern Manitoba. *Journal of Hydrology*, **196**, 310–323.

Pryet, A., Domínguez, C., Tomai, P., Chaumont, C., d’Ozouville, N., Villacís, M., and Violette, S. (2012) Quantiﬁcation of cloud water interception along the windward slope of Santa Cruz Island, Galapagos (Ecuador). *Agricultural and Forest Meteorology*, **161**, 94–106.

Pryor, S. and Barthelmie, R. (2005) Liquid and Chemical Fluxes in Precipitation, Throughfall and Stemflow: Observations from a Deciduous Forest and a Red Pine Plantation in the Midwestern U.S.A. *Water Air and Soil Pollution*, **163**, 203-227

Pukjaloon, V., Pongboun, K. and Rouysungnern, S. (1984) Rainfall interception by *Eucalyptus camaldulensis*. In A. Nalanpoon, P. Thitirojanawat and P. Tippharaot (Eds.), *Abstract of Research Works in Watershed Management in Thailand* (#16). Bangkok, Thailand: Forest Environment Research and Development Division, Forest Research Office, Royal Forest Department.

Qiang, S.Q., and Zhi, C.L. (2000) Characteristics of precipitation and forest stemflow of Dongling Mountainous area. *Acta Ecologica Sinica*, **2000**(1), Q948.112.

Radzi Abas, M., Ahmad-Shah, M., and Nor Awang, M.  (1992) Fluxes of ions in precipitation, throughfall and stemflow in an urban forest in Kuala Lumpur, Malaysia. *Environmental Pollution*, **75**, 209-213.

Rapp, M. and Ibrahim, M. (1978) Égoutement, écoulement et interception des précipitations par un peuplement de *Pinus pinea* L. *Oecologia Plantarum*, **3**, 271-284.

Reid, L. and Lewis, J. (2009) Rates, timing, and mechanisms of rainfall interception loss in a coastal redwood forest. *Journal of Hydrology*, **375**, 459-470.

Reynolds, E.R.C. and Henderson, C.S. (1967) Rainfall interception by beech, larch and Norway spruce. *Forestry*, **40**, 165-184.

Rodrigo, A., Avila, A., and Roda, F. (2003) The chemistry of precipitation, throughfall and stemflow in two holm oak (Quercus ilex L.) forests under a contrasted pollution environment in NE Spain. *Science of the Total Environment*, **305**, 195-205

Rogerson, T. and Byrnes, W. (1968) Net rainfall under hardwoods and red pine in central Pennsylvania. *Water Resources Research*, **4**, 55-57

Rothacher, J. (1963) Net precipitation under a Douglis fir forest. *Forest Science*, **9**, 423-429

Roth-Nebelsick, A., Ebner, M.,  Miranda, T., Gottschalk, V., Voigt, D., Gorb, S., Stegmaier, T., Sarsour, J., Linke, M., and Konrad, W. (2012) Leaf surface structures enable the endemic Namib desert grass (Stipagrostis sabulicola) to irrigate itself with fog water. *Journal of the Royal Society*, **9**, 1965-1974.

Sadeghi, S.M.M., Attarod, P., Van Stan, J.T. and Pypker, T.G. (2016) The importance of considering rainfall partitioning in afforestation initiatives in semiarid climates: A comparison of common planted tree species in Tehran, Iran. *Science of the Total Environment*, **568**, 845-855.

Sadeghi, S.M.M., Van Stan, J.T., Pypker, T.G., and Friesen, J. (2017) Canopy hydrometeorological dynamics across a chronosequence of a globally invasive species, *Ailanthus altissima* (Mill., tree of heaven). *Agricultural and Forest Meteorology*, **240**, 10-17.

Saffigna, P.G., Tanner, C.B., and Keeney, D.R. (1976) Non-uniform infiltration under potato canopies caused by interception, stemflow, and hilling. *Agronomy Journal*, **68**, 337-342.

Sakai, H., Sengoku, T., Hara, M., Morisawa, T., Taoda, H., Iwamoto, K., Arai, K. and Ozawa, T. (2004) Monitoring of acidic precipitaion’s effects on forest ecosystems – Rainwater chemistry in a subalpine coniferous forest at Mt. Ontake, Nagano Prefecture, Central Japan. *Belletin of FFPRI*, **3**, 297-317.

San Jose, J.J. and Montes, R. (1992) Rainfall partitioning by a semideciduous forest grove in the savannas of the Orinoco Llanos, Venezuela. *Journal of Hydrology*, **132**, 249-262.

Sansoulet, J., Cabidoche, Y.M., and Cattan, P. (2007) Adsorption and transport of nitrate and potassium in an Andosol under banana (Guadeloupe, French West Indies). *European Journal of Soil Science*, **58**, 478-489.

Santa Regina, I., and Tarazona, T. (2000) Nutrient return to soil through litterfall and throughfall under beech and pine stands of Sierra de la Demanda, Spain. *Arid Soil Research and Rehabilitation*, **14**, 239-252.

Schmull, M., Hauck, M., Vann, D.R., Johnson, A.H. and Runge, M. (2002) Site factors determining epiphytic lichen distribution in a dieback-affected spruce-fir forest on Whiteface Mountain, New York: stemflow chemistry. *Canadian Journal of Botany*, **80**, 1131-1140.

Schooling, J.T., and Carlyle-Moses, D.E. (2015) The influence of rainfall depth class and deciduous tree traits on stemflow production in an urban park. *Urban Ecosystems*, **18**, 1261-1284.

Schooling, J.T., Levia, D.F., Carlyle-Moses, D.E., Downtin, A.L., Brewer, S.E., Donkor, K.K., Borden, S.A. and Grzybowski, A.A. (2017) Stemflow chemistry in relation to tree size: A preliminary investigation of eleven urban park trees in British Columbia, Canada. *Urban Forestry & Urban Greening*, **21**, 129-133.

Schroth, G., Elias, M.E.A., Uguen, K., Seixas, R., and Zech, W. (2001) Nutrient fluxes in rainfall, throughfall and stemflow in tree-based land use systems and spontaneous tree vegetation of central Amazonia. *Agriculture Ecosystems and Environment*, **87**, 37-49.

Serrato, F.B., and Diaz, A.R. (1998) A simple technique for measuring rainfall interception by small shrub: “interception flow collection box”. *Hydrological Processes*, **12**, 471-481.

Silva, I.C., and Rodríguez, H.G. (2001) Interception loss, throughfall and stemflow chemistry in pine and oak forests in northeastern Mexico. *Tree Physiology*, **21**, 1009-1013.

Singer, A., Ganor, E., Fried, M., and Shamay, Y. (1996) Throughfall deposition of sulfur to a mixed oak and pine forest in Israel. *Atmospheric Environment*, **30**, 3881-3889.

Singh, R.P. (1987) Rainfall interception by *Pinus wallichiana* plantation in temperate region of Himachal Pradesh, India. *Indian Forester*, **113**, 559-566.

Sinun, W., Meng, W.W., Douglas, I., and Spencer, T. (1992) Throughfall, stemflow, overland flow and throughflow in the Ulu Segama rain forest, Sabah, Malaysia. *Philosophical Transactions: Biological Sciences*, **335**, 389-395.

Skau, C.M. (1964) Interception, throughfall, and stemflow in Utah and alligator juniper cover types of Northern Arizona. *Forest Science*, **10**, 283-287.

Slayter, R.O. (1965) Measurements of precipitation interception by an arid zone plant community (*Acacia anuera* F. Muell.). *Arid Zone Research*, **25**, 181-192.

Songwattana, V., Rouysungnern, S. and Onnom, V. (1988) Rainfall interception by some tree species in forest plantation. In A. Nalanpoon, P. Thitirojanawat and P. Tippharaot (Eds.), *Abstract of Research Works in Watershed Management in Thailand* (#34). Bangkok, Thailand: Forest Environment Research and Development Division, Forest Research Office, Royal Forest Department.

Specht, R.L. (1957) Dark Island heath (Ninety-mile Plain, South Australia). IV. Soil moisture patterns produced by rainfall interception and stem-flow. *Australian Journal of Botany*, **5**, 137-150.

Spittlehouse (1998) Rainfall interception in young and mature conifer forests in British Columbia. In *Proceedings 23rd Conference on Agricultural and Forest Meteorology* (pp. 171-174). Boston, MA, USA: American Meteorological Society.

Sridhar, K. and Karamchand, K. (2009) Diversity of ware-borne fungi in stemflow and throughfall of tree canopies in India. *Sydowia*, **61**, 347-364

Srivastava, A.K., and Ambasht, R.S. (1994) Nitrogen deposition in *Casuarina equisetifolia* (Forst.) plantation stands in the dry tropics of Sonbhadra, India. *Forest Ecology and Management*, **70**, 341-348.

Staelens, J. (2006) Spatio-temporal patterns of throughfall water and ion deposition under a dominant beech tree (*Fagus sylvatica* L.) in relationship to canopy structure. Doctoral thesis, Ghent University.

Steinhardt, U. (1978) Uuntersuchungen ueber den Wasser- und Naehrstoffhaushalt eines andinen Wolkenwaldes in Venezuela. *Goettinger Bodenkundliche Berichte*, **56**, 185 p.

Takahashi, T., Nagahori, C., Mongolsawat, C., and Losirikul, M. (1983) Runoff and soil loss. In K. Kyuma and C. Pairintra (Eds.) *Shifting Cultivation - An Experiment at Nam Phron, Northeast Thailand* (pp. 84-109). Bangkok, Thailand: Ministry of Science Technology and Energy.

Tanaka, N., Tantasirin, C., Kuraji, K., Suzuki, M., and Tangtham, N. (2005) Inter-annual variation in rainfall interception at a hill evergreen forest in Northern Thailand. *Bulletin of Tokyo University Forests*, **113**, 11-44.

Tangtham, N. (1973) Ecosystem of Kog-Ma Watershed Research Station. *Kog-Ma Watershed Research Bulletin* (12). Bangkok, Thailand: Kasetsart University.

Tani, M., Nik, A.R., Yasuda, Y., Noguchi, S., Syamsuddin, S.A., Sahat, M.M., and Takanashi, S. (2003) Long-term estimation of evapotranspiration from a tropical rain forest in peninsular Malaysia. In S. Franks, G. Blsochl, M. Kumagai, K. Mushiake, and D. Rosbjerg (Eds.) *Water Resources System Water Availability and Global Change* (pp. 267-274). IAHS Publication 280.

Taniguchi, M., Tsujimura, M. and Tanaka, T. (1996) Significance of stemflow in groundwater recharge. 1: Evaluation of the stemflow contribution to recharge using a mass balance approach. *Hydrological Processes*, **10**, 71-80.

Thurow, T., Blackburn, W., Warren, S., and Taylor, C. Jr. (1987) Rainfall interception by midgrass, shortgrass, and live oak mottes. *Allen Press and Society for Range Management*, **40**, 455-460

Tian, D., Sheng, L., He, B. and Xu, H. (1994) A study on hydrological effect of artificial disturbance in a Chinese fir plantation ecosystem. In X. Zhou (Ed.) *Studies on Forest Ecosystems* (pp. 384-393). Harbin, China: Northeast Forestry University Press.

Toba T. and Ohta T. (2005) An observational study of the factors that influence interception loss in boreal and temperate forests. *Journal of Hydrology*, **313**, 208–220

Tobón, C., Sevink , J. and Verstraten, J.M. (2004) Solute fluxes in throughfall and stemflow in four forest ecosystems in northwest Amazonia. *Biogeochemistry*, **70**, 1-25.

Tsakov, H., and Alexandrov, A. (2005) Growth of *Robinia pseudoacacia* L. on a reclaimed terrain in Bulgaria studied over a period of climatic anomalies. *Folia Oecologia*, **32**, 1-42.

Turvey, N.D. (1974) Water in the nutrient cycle of a Papuan rain forest. *Nature*, **251**, 414-415.

Twilley, R. and Chen, R. (1998) A water budget and hydrology model of a basin mangrove forest in Rookery Bay, Florida. *Marine and Freshwater Research*, **49**, 309– 323.

Ubarana, V.N. (1996) Observations and modelling of rainfall interception at two experimental sites in Amazonia. Chapter 8 in J.H.C. Gash, C.A. Nobre, J.M. Roberts, and R.L. Victoria (Eds.) *Amazonian Deforestation and Climate* (pp. 151-162). Institute of Hydrology.

Uyttendaele, G. and Iroumé, A. (2002) The solute budget of a forest catchment and solute fluxes within a Pinus radiata and secondary native forest site, southern Chile. *Hydrological Processes*, **16**, 2521-2536.

Valente, F., David, J., and Gash J. (1997) Modelling interception loss for two sparse eucalypt and pine forests in central Portugal using reformulated Rutter and Gash analytical models. *Journal of Hydrology*, **190**, 141-162

Van Cleve, K., Oliver, L., Schlentner, R., Viereck, L., and Dyrness, C. (1983) Productivity and nutrient cycling in taiga forest ecosystems. *Canadian Journal of Forest Research*, **13**, 747–766.

Van Stan, J.T. (2012) Controls and dynamics of canopy-derived dissolved organic matter from co-dominant broadleaved deciduous canopies to the soil of a temperate catchment in the northeastern United States. Doctoral thesis, University of Delaware.

Van Stan, J.T., Wagner, S., Guillemette, F., Whitetree, A., Lewis, J., Silva, L., and Stubbins, A. (2017) Temporal dynamics in the concentration, flux, and optical properties of tree-derived dissolved organic matter (tree-DOM) in an epiphyte-laden oak-cedar forest. *Journal of Geophysical Research-Biogeosciences*, under review.

Veneklaas, E. and Van Ek, R. (1990) Rainfall interception in two tropical montane rain forests, Colombia. *Hydrologic Processes*, **4**, 311–326

Vernimmen, R., Bruijnzeel, L., and Romdoni, A. (2007) Rainfall interception in three contrasting lowland rain forest types in Central Kalimantan, Indonesia. *Journal of Hydrology*, **340**, 217–232

Verry, E. and Timmons, D. (1977) Precipitation nutrients in the open and under two forests in Minnesota. *Canadian Journal of Forest Research*, **7**, 112-119

Viereck. L., Dyrness, C., Van Cleve, K., and Foote, M. (1983) Vegetation, soils, and forest productivity in selected forest types in interior Alaska. *Canadian Journal of Forest Research*, **13**, 703–720

Viville, D., Biron, P., Granier, A., Dambrine, E., and Probst, A. (1993) Interception in a mountainous declining spruce stand in the strengbach catchment (Vosges, France). *Journal of Hydrology*, **144**, 273–282

Voigt, G. (1960) Distribution of rainfall under forest stands. *Forest Science*, **6**, 2-9

Weaver, P. (1972) Cloud moisture interception in the Luquillo mountains of Puerto Rico. *Caribbean Journal of Science*, **12**, 129–144

Wei, X. and Zhou, X. (1991) Hydrological characteristics of oak forests. In X. Zhou (Ed.) *Studies on Forest Ecosystems* (pp. 332-345). Harbin, China: Northeast Forestry University Press.

West, N. and Gifford, G. (1976) Rainfall interception by cool desert shrubs. *Journal of Range Management*, **29**, 171–172

Wheater, H., Langan, S., and Miller, J. (1987) The determination of hydrological flow paths and associated hydrochemistry in forested catchments in central Scotland. *International Association of Hydroly Science Publication*, **167**, 433–449

White, I., Falkland, A., Metutera, T., Metai, E., Overmars, M., Perez, P., and Dray, A. (2007) Climatic and human influences on groundwater in low atolls. *Vadose Zone Journal,* **6**, 581–590

Whitford, W., Anderson, J., and Rice, P. (1997) Stemflow contribution to the “fertile island” effect in creosotebush, *Larrea tridentata*. *Journal of Arid Environments*, **35**, 451–457.

Widiyono, W. (2010) Inventarisasi jenis-jenis tumbuhan dan kesesuaian lahan untuk konservasi daerah tangkapan sumber mata air ‘wetihu’ desa baudaok kecamatan tasfifeto timur - Belu. *J Tek Ling*, **11**, 353-361.

Williams, C., Silins, U., and Wagner, M. (2012) Throughfall, Stemflow, and Rainfall Interception in a Severely Burned Subalpine Forest. American Geophysical Union Fall Meeting, H33E-1375.

Wilm, H. and Niederhof, C. (1941) Interception of rainfall by mature lodgepole-pine. *Earth and Space Science News*, **22**, 660-665

Witthawatchutikul, P., and Tangtham, N. (1987) Rainfall intercepted in logged-over dry evergreen forest at Huay Ma Fuang, Rayong Province. In A. Nalanpoon, P. Thitirojanawat, and P. Tippharot (Eds.) *Abstract of Research Works in Watershed Management in Thailand* (pp. 38). Bangkok, Thailand: Forest Environment Research and Development, Royal Forest Department.

Xiao, Y., Chen, L., and Yu, X. (2007) Characteristics of precipitation redistribution and stemflow in *Quercus acutissima* mixed forest in Miyun, Beijing. *Journal of Northeast Forestry University*, **22**, 16-18.

Yan, J., Zhou, G., Zhang, D., and Wang, X. (2003) Spatial and temporal variations of some hydrological factors in a climax forest ecosystem in the Dinghushan region. *Acta Ecologica Sinica*, **23**, 2359-2366.

Young, J., Evans, R., and  Easi, D. (1984) Stem flow on western juniper (Juniperus occidentalis) trees. *Weed Science*, **32**, 320–327.

Zabret, K., and Šraj, M. (2015) Rainfall interception by deciduous and coniferous trees in an urban area. European Geosciences Union General Assembly, **17**, 2015-745.

Zeng, Q. (1994) Hydrologic cycling of tropical forest ecosystems in Jianfengling Hainan island, Long Term Research on China’s Forest Ecosystems. In X. Zhou (Ed.) *Studies on Forest Ecosystems* (pp. 413-429). Harbin, China: Northeast Forestry University Press.

Zhang, F., Zhang, J., Zhang, H., Ogura, N. and Ushikubo, A. (1996) Chemical composition of precipitation in a forest area of Chongqing, Southwest China. *Water Air and Soil Pollution*, **90**, 407-415.

Zhang, J., Van Meerveld, I., Waterloo, M., and Bruijnzeel, L. (2015) Typhoon Haiyan's Effects on Interception Loss from a Secondary Tropical Forest near Tacloban, Leyte, the Philippines. American Geophysical Union Fall Meeting, H13Q-06.

Zhongjie, S., Wang, Y., Xu, L., Xiong, W., Yu, P., Gao, J., and Zhang, L. (2010) Fraction of incident rainfall within the canopy of a pure stand of Pinus armandii with revised Gash model in the Liupan Mountains of China. *Journal of Hydrology*, **385**, 44-50

Zhou, M. (2003) Hydrological studies in the Inner Mongolian Forests. Doctoral thesis. Inner Mongolian Agriculture University.
